# TAP: a targeted clinical genomics pipeline for detecting transcript variants using RNA-seq data

**DOI:** 10.1186/s12920-018-0402-6

**Published:** 2018-09-10

**Authors:** Readman Chiu, Ka Ming Nip, Justin Chu, Inanc Birol

**Affiliations:** 1 Canada’s Michael Smith Genome Sciences Centre, BC Cancer Agency, 100-570 West 7th Ave, Vancouver, BC V5Z 4S6 Canada; 2 Department of Medical Genetics, The University of British Columbia, Vancouver, BC Canada

**Keywords:** RNA-seq, Transcriptome assembly, Clinical genomics, Gene fusion, Alternative splicing, Internal tandem duplication, Partial tandem duplication, Acute myeloid leukemia

## Abstract

**Background:**

RNA-seq is a powerful and cost-effective technology for molecular diagnostics of cancer and other diseases, and it can reach its full potential when coupled with validated clinical-grade informatics tools. Despite recent advances in long-read sequencing, transcriptome assembly of short reads remains a useful and cost-effective methodology for unveiling transcript-level rearrangements and novel isoforms. One of the major concerns for adopting the proven *de novo* assembly approach for RNA-seq data in clinical settings has been the analysis turnaround time. To address this concern, we have developed a targeted approach to expedite assembly and analysis of RNA-seq data.

**Results:**

Here we present our Targeted Assembly Pipeline (TAP), which consists of four stages: 1) alignment-free gene-level classification of RNA-seq reads using BioBloomTools, 2) *de novo* assembly of individual targets using Trans-ABySS, 3) alignment of assembled contigs to the reference genome and transcriptome with GMAP and BWA and 4) structural and splicing variant detection using PAVFinder. We show that PAVFinder is a robust gene fusion detection tool when compared to established methods such as Tophat-Fusion and deFuse on simulated data of 448 events. Using the Leucegene acute myeloid leukemia (AML) RNA-seq data and a set of 580 COSMIC target genes, TAP identified a wide range of hallmark molecular anomalies including gene fusions, tandem duplications, insertions and deletions in agreement with published literature results. Moreover, also in this dataset, TAP captured AML-specific splicing variants such as skipped exons and novel splice sites reported in studies elsewhere. Running time of TAP on 100–150 million read pairs and a 580-gene set is one to 2 hours on a 48-core machine.

**Conclusions:**

We demonstrated that TAP is a fast and robust RNA-seq variant detection pipeline that is potentially amenable to clinical applications. TAP is available at http://www.bcgsc.ca/platform/bioinfo/software/pavfinder

**Electronic supplementary material:**

The online version of this article (10.1186/s12920-018-0402-6) contains supplementary material, which is available to authorized users.

## Background

Advances in second-generation sequencing technologies ushered in the modern era of personalized medicine [1]. In cancer, mutations revealed by clinical sequencing have been shown to be vitally useful in achieving better subtype classification, charting appropriate treatment regimens, and identifying novel drug targets [2–4]. One of the well-studied examples is acute myeloid leukemia (AML), for which prognosis and treatment strategies depend on the detection of a wide spectrum of mutations: *FLT3* internal tandem application (ITD), *MLL* partial tandem duplication (PTD), *NPM1* insertion, *CEBPA* insertion/deletions (indels), and gene fusions *PML-RARA*, *RUNX1-RUNX1T* and *CBFB-MYH11*, among others [5].

While the cost of sequencing has decreased dramatically since its introduction, translating whole-genome sequencing methods to the clinical domain remains a challenge due to their sample amount and quality, coverage depth, and turnaround time requirements [6]. With its lower cost and input sample requirements, and faster turnaround times, RNA sequencing (RNA-seq) offers an attractive alternative. Although in the research domain it is primarily used to unveil altered gene expression levels, RNA-seq is increasingly used to capture expressed genomic anomalies such as single nucleotide variants (SNVs) and aberrant transcript structures [7, 8].

Important sequence-based disease markers typically are SNVs, but long-range rearrangements or structural variants (SVs) are also being increasingly appreciated for their important roles in pathogenesis [9]. RNA-seq read sequences allow SNVs and short indels to be readily identified, but SV detection with short reads requires more complex analysis and algorithms. Although the recent development of long-read sequencing technologies has shown promise in facilitating the reconstruction of full-length transcripts and novel isoforms [10, 11], their application in clinical settings remains to be fully explored and assessed for reliability and throughput considerations.

Current state-of-the-art SV detection tools, such as TopHat-Fusion [12] and deFuse [13], discover SVs through interrogating alignments of reads to the reference genome, but ambiguous alignments of short reads limit the sensitivity and specificity of these methods. *De novo* RNA-seq assembly reconstructs long transcript sequences without relying on alignments of reads to a reference genome, and thus it is widely used in profiling transcriptomes of non-model organisms, reconstructing transcript structures, and detecting novel isoforms [14–18]. Because longer sequences tend to have lower alignment ambiguity than short sequences, SV detection based on alignments of assembled transcripts (instead of short reads) to a reference genome have been shown to be successful in cancer studies [19, 20]. However, analysis of deeply sequenced human transcriptomes remains very resource-intensive, and therefore may not meet the constraints in the clinical domain.

An alternative to analyzing entire genomes or transcriptomes is to focus on target gene sets (or gene panels) that are most relevant for specific diseases [21, 22]. This is effective because for many diseases, in particular cancers, there are many clinically relevant genes to help with disease classification or with the selection of treatment strategies [23]. Here we propose a targeted approach on RNA-seq data analysis called TAP (for Targeted Assembly Pipeline) using *de novo* assembly for variant identification. TAP offers functionality akin to using data from gene panels, and offers the benefits of a robust sequencing protocol coupled with the flexibility of selecting the genes of interest after data generation, as the clinical question might dictate.

## Implementation

TAP detects SVs in four stages described below. It also summarizes other pertinent information, such as the extent of reconstruction of all the targeted genes, and a compilation of all the reconstructed splice junctions (novel or annotated), and their supporting read counts.

### Alignment-free extraction of reads for gene targets

**Fig. 1:**
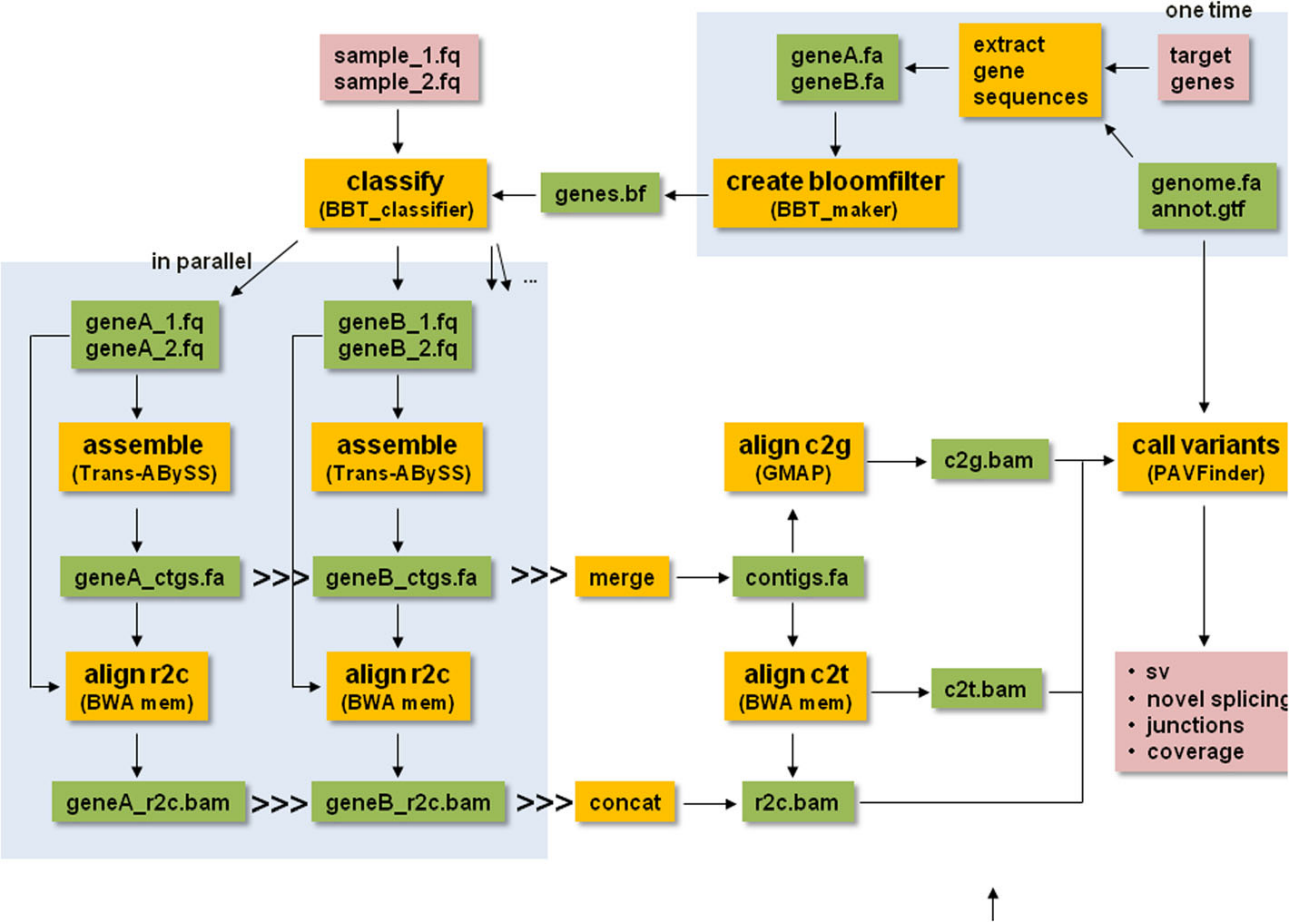
TAP Pipeline. A Bloom filter is generated from reference transcript sequences of a target list and then applied on full transcritpome RNA-seq sequences to extract gene-specific read pairs. Reads classified to each target are segregated into separate bins and assembled using two *k*-mer values independently in parallel. Contigs from each *k*-mer assembly of each gene are merged and extracted reads are aligned to them (r2c). Gene-level assemblies are combined into a single file and aligned to the genome (c2g) and transcriptome (c2t). PAVFinder uses the c2g and c2t alignments together with contig sequences and annotation (reference sequences and gene models) to identify structural variant and novel splicing events. r2c alignments are used for determining event support and coverage estimation

The first step of TAP (Fig. 1) is to classify and segregate whole-sample RNA-seq reads into bins corresponding to specific gene targets. Instead of using alignment-based approaches for this purpose, we chose to use a novel multi-index Bloom filter data structure implemented within BioBloomTools (BBT v2.1.0), which is able to achieve sequence classification at comparable accuracy to alignment-based methods, but in a much faster and memory-efficient manner [24]. The inputs to this sequence extraction step are RNA-seq read pairs and transcript sequences of a list of target genes. BBT utilizes a set of five spaced seeds with an allowed miss of two spaced seeds (parameter -a) per *k*-mer frame evaluated. This allows BBT to better tolerate sequencing errors and variants, and achieve high sensitivity whilst maintaining high specificity. Further, it extracts read pairs when at least one of the pairs is classified as hitting one of the target genes (flag -i), capturing sequences that represent novel splice variants and gene fusions.

### *De novo* reconstruction of transcript sequences

Bins of read pairs belonging to individual genes are assembled independently in parallel using Trans-ABySS (v1.5.4) [14]. *De novo* assembly is used to reconstruct variant breakpoint-spanning sequences from short reads, and Trans-ABySS has been shown to be successful in capturing such events in various genomic and transcriptomic studies [25–33]. To reconstruct transcripts with a range of expression levels and sequence complexity, Trans-ABySS uses a set of overlap lengths (*k*-mer sizes). Typically, a low *k-*mer size would be more sensitive to read-to-read overlaps, helping reconstruct low expressed transcripts, while a high *k-*mer size would be more specific to resolve low complexity sequences.

### Alignment of assembled transcripts and extracted reads

Since transcriptomic rearrangements can be complicated and may lead to erroneous alignments, alignments to both reference genome and transcriptome are used in TAP to increase accuracy and sensitivity. Assembled contig sequences are aligned to the reference genome and the reference transcriptome using GMAP (v2014-12-28) [34] and the BWA-MEM algorithm of the BWA package (v0.7.12) [35], respectively. Concurrently, extracted reads are aligned to the assembled contigs to provide support evidence and read counts of SV calls.

### Detection of structural variants

**Fig. 2:**
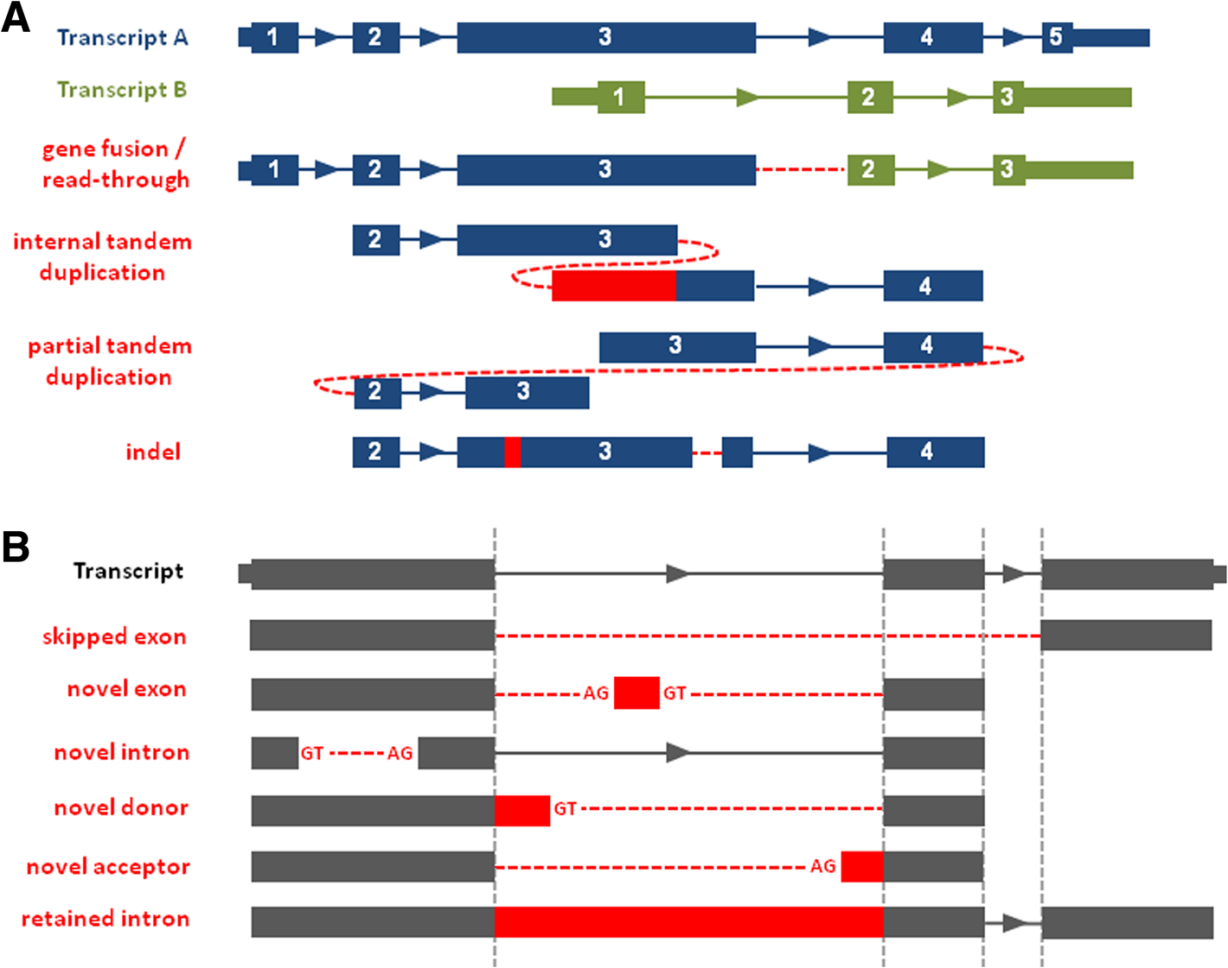
PAVFinder detects both (**a**) structural rearrangements and (**b**) novel splicing variants. Numbers indicate reference transcript exon numbers. Dotted red lines represent novel adjacencies (joining between non-adjacent transcript sequences) and red blocks represent novel sequences. For splicing variants, canonical splice site motifs are indicated as they are checked for calling potential novel splicing events. Dotted vertical lines depict algorithm for detecting novel splicing variants by aligning contig sequences against annotated gene model

As a key module within TAP, we developed PAVFinder (Post Assembly Variant Finder, v0.4.2) to deduce variants from the split or gapped alignments of contigs to the references (Fig. 2a). Based on several criteria (Additional file 1: Table S1, S2), such as the alignment orientation of chimeric sequence fragments, PAVFinder classifies events such as gene fusions, read-throughs, ITDs, PTDs, indels and repeat number changes. It also reports novel splicing events, such as exon skipping, novel exons, novel introns, retained introns, and novel splice donor and acceptor sites by comparing contig-to-genome alignments to reference gene models (Fig. 2b).

## Results

### Assessment of the performance of BBT in sequence classification

TAP analyses the sequences selected by BBT. To assess BBT’s performance in sequence classification at different sequencing depths, we simulated Illumina (San Diego, CA) reads with depth of coverage ranging from 10× (229,800 read pairs) to 100× (2,303,019 read pairs) in increments of 10 using pIRS (v1.1.1) [36] from a gene set composed of 580 COSMIC (v77) genes [37] (targets) and an equal number of non-COSMIC genes randomly selected from RefSeq [38]. The non-COSMIC genes were included to mimic non-target genes in the transcriptome. We chose to use pIRS over other RNA-seq simulators because of its simplicity to simulate different read depths and its provision of read-origin information, which readily enables calculation of classification accuracy. We compared the performance of BBT on the COSMIC set against alignment-based classification using BWA-MEM (v0.7.12) to observe that BBT slightly outperformed BWA-MEM in overall sensitivity (BBT 99.9% versus BWA-MEM 98.1%) and both methods show comparable specificity (BBT 99.2% versus BWA-MEM 99.9%) (Additional file 1: Figure S1). However, on a per-gene basis, we found BBT to outperform BWA-MEM in 115 genes, while BWA-MEM outperformed BBT in 66 genes (the remaining 399 are in a virtual tie with their F1 scores within one standard deviation of each other) (Additional file 1: Figure S2). This trend is reverted for the software parameterization used above when we increased the substitution-error rate from 0.37% (default profile of the experimental data) to 1% in the simulation step. For this unusually high error rate, BWA-MEM outperformed BBT in 303 genes, whereas BBT was superior in only 104. The actual overall difference in absolute performance metrics is, however, negligible (within 0.1% in most coverage depths) (Additional file 1: Figure S3). In terms of computation performance, BBT runs faster than BWA-MEM, and scales much better with increasing read depths (Additional file 1: Figure S1).

### Assessment of the performance of PAVFinder

**Fig. 3:**
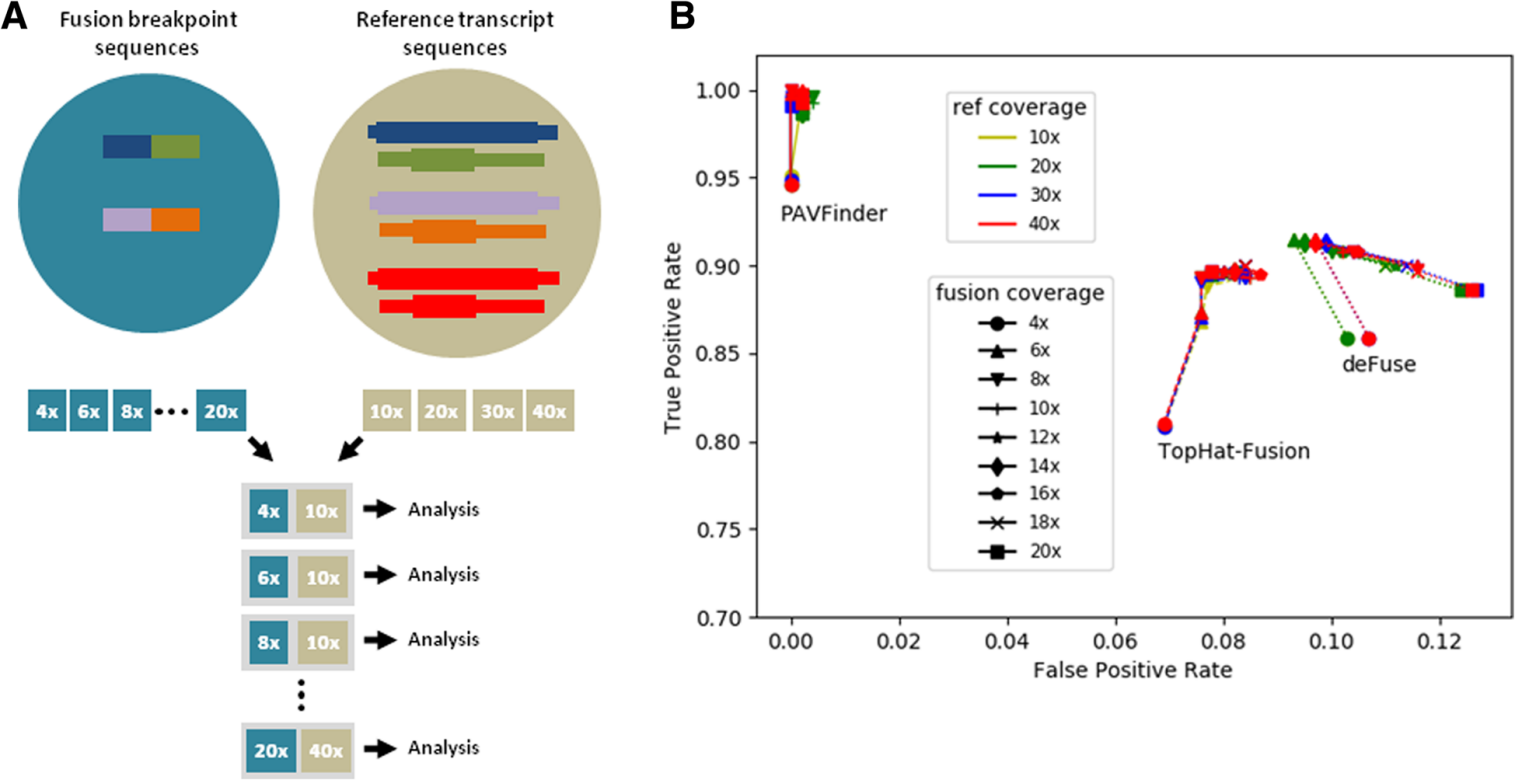
Simulation experiment to assess PAVFinder fusion calling performance in relation to sequencing coverage and other software. **a**. Design of experiment: reads simulated from fusion breakpoints and corresponding reference transcript sequences at different read depths are combined to simulate the titration series. **b**. Receiver Operating Characteristic (ROC) plots of PAVFinder, Tophat-Fusion [12], and deFuse [13] on 448 fusion events reported on TCGA data [39]

We investigated the fusion-calling performance of PAVFinder in relation to sequencing depth, and compared that with two well-established methods in the field [12, 13]. From a published list of gene fusions reported from TCGA RNA-seq experiments [39], 448 “tier-1” (highest level of confidence in the study), inter-chromosomal, and in-frame events with defined breakpoint locations were selected to simulate a titration series consisting of varying sequencing coverages (4× to 20×, increments of 2) of the breakpoint sequences (250 base pair (bp) mean simulation insert size, upstream and downstream of the breakpoint), mixed with whole transcript reference sequences of the fusion genes (803, discounting redundant gene partners) together with a similar number (776) of randomly-selected non-fusion transcripts as background at four different coverage depths (10× to 40×, increments of 10) (Fig. 3a). We used pIRS [36] to simulate 100 bp Illumina reads with a mean insert size of 250 bp for each coverage combination.

For benchmarking, only events with at least four breakpoint-spanning reads were considered for comparison. A true positive is scored when partners in a detected gene fusion event correspond to one of the 448 input gene pairs. In this experiment, PAVFinder shows high sensitivity (about 95%) at fusion coverage depth of 4×, and this performance reaches 100% at higher fusion coverage depths. The false positive rate remains less than 0.5% throughout the sequencing coverage depths evaluated. When compared with the other tools, PAVFinder has the highest sensitivity and specificity (Fig. 3b).

### Assessment of the performance of TAP on real data

The Leucegene Project [40] made available 437 publicly accessible RNA-seq datasets analyzed in several published studies [41–44]. We leveraged these data (Table 1) for evaluating the performance of TAP due to the wide spectrum of structural variants that are clinically-relevant biomarkers offered by this disease. Using 580 COSMIC cancer genes [37] as our target gene set, we examined fusions and read-throughs of

- the core-binding factor (CBF) cohort [42], which carries either the *CBFB-MYH11* or *RUNX1-RUNX1T1* fusion;
- the *NUP98-NSD1* cohort [43], which carries the *NUP98-NSD1* fusion; and
- the *MLL* fusion (*MLL*-F) cohort, which carries *MLL* (a.k.a. *KMT2A*) fused with different partners [41].

**Table 1:** Leucegene AML samples analyzed in this study

Cohort	Number of samples analyzed	GEO Accession
core-binding factor (CBF)	46	GSE62190
		GSE67039
		GSE52656
*NUP98-NSD1*	7	GSE49642
		GSE67039
		GSE52656
*MLL*-fusion (*MLL*-F)	31	GSE52656
		GSE49642
		GSE67039
		GSE52656
*CEBPA*	12	GSE67039
		GSE52656
		GSE49642
		GSE62190
		GSE66917
*MLL*-PTD	1	GSE67039

We screened a multitude of samples for *MLL*-PTD as the sample identities of the *MLL*-PTD cohort were not disclosed in any of the Leucegene publications. Furthermore, we processed samples from a *CEBPA* cohort to assess TAP’s ability to detect short indels. For all the processed samples, we also look for the important AML variants *FLT3*-ITD and *NPM1* insertion. Finally, we identified several aberrant splicing events reported in the literature [45, 46] that have potential implications in AML, and checked whether we can detect them in any of the samples we analyzed.

### Fusions

We processed 46 RNA-seq samples of the CBF cohort, of which 26 carry the *CBFB*-*MYH11* fusion (inv(16)), and 20 carry the *RUNX1*-*RUNX1T1* fusion (t(8;21)). TAP was successful in detecting all of the fusion events, in agreement with the literature [30]. Two of the *CBF*-*MYH11* cases (03H095 and 12H042) do not have breakpoints at exon boundaries: one presents four extra amino acids at the junction, and the other has a breakpoint internal to the *MYH11* exon, both of which nevertheless produce in-frame chimeric transcripts. The PAVFinder module was configured by default to restrict fusion breakpoints to exon boundaries. When this option was turned off, the *CBF*-*MYH11* fusion were identified and reported in TAP. PAVFinder also detected the *NUP98*-*NSD1* fusion in all seven AML samples known to contain the fusion event.

We also processed 31 samples of the *MLL*-F cohort, which contains *MLL* fusions involving different partners: *CASC5* (1), *ENL* (*MLLT1*, 4), *ELL* (3), *GAS7* (1), *SEPT9* (2), *MLLT* (9), *MLLT4* (8), *MLLT6* (1) and *MLLT10* (2) (numbers in brackets indicate number of samples in each case). TAP could detect all nine types of MLL fusions in 30 out of the 31 samples. The only sample we failed to detect any fusion events was 04H080, which was reported to carry the fusion *MLL*-*MLLT3*. It was noted in the publication that the fusion was detected by only two reads. We note that the sample contains only 23 million read pairs, only about a quarter of the average size of the other samples. To troubleshoot, we ran the original tool that was used to detect the fusion, TopHat-fusion [12], and still was unable to detect the event.

To benchmark PAVFinder’s relative performance in gene fusion detection, we also processed both the extracted and entire read sets of all the *CBF*-*MYH11*, *NUP98*-*NSD1*, and *MLL*-F cohorts with TopHat-Fusion [12] and deFuse [13]. Using mostly default parameters (Additional file 1: Table S5), except with the requirement of at least four reads spanning a breakpoint, TopHat-Fusion failed to report eight *MLL*-F and one *CBF*-*MYH11* fusions in the reads extracted by BBT. Using the entire read set did not change the results. In two *CBF*-*MYH11* samples, deFuse failed to report the correct fusion in the extracted read set as it had misaligned the *MYH11* segment to *Nde1*. Interestingly, this misalignment was not observed when the entire read set was used. In one *MLL*-F sample, deFuse failed to detect the *MLL* fusion in both the extracted and the entire read sets. The samples for which TopHat-Fusion and deFuse missed calling the expected fusion events using the extracted reads were not the same samples, suggesting that it is not the sequence extraction step that causes the false-negatives.

In addition to the signature AML events from the Leucegene study, TAP reported further fusion calls. We assessed these events for their validity by searching for previous reports in the literature. Out of a total of 47 events, about half of them (28) have been reported before (Additional file 1: Figure S4). Notable cases include *ETV6*-*NTRK3*, a well-known driver detected in various cancer types including AML [47, 48], and *TFG*-*ADGRG7*, a known event reported in healthy individuals [49]. Of the events without any publication reference, one of the partner genes is often found in another fusion event reported in the literature. Examples include fusions involving *DDX5*, *CXCR4*, *KLF2*, and *UBC*. Fusions in this last category usually exhibit low expression levels, and are apparently promiscuous regarding their fusion partners. Marincevic-Zuniga et al. [50] “blacklisted” these genes, and filtered them out in their detection pipeline, suggesting that although these fusions may indeed be bona fide, their biological significance is dubious or unknown. Amid these “noise” events, however, one novel fusion, *PHKB*-*ATTC,* is potentially promising (46 spanning read support) and biologically functional (in-frame fusion of the 5′ of *PHKB* to the 3’of *ATTC*), while another, *FCGR2C*-*FCGR2A*, is most likely a false positive as a result of mis-assembly due to extensive sequence similarity.

Read-throughs are chimeric transcripts resulting from splicing of two adjacent genes on the same coding strand. They have been found in both normal and neoplastic tissues. An example is *SLC45A3*-*ELK4*, which has been detected before in prostate cancer [51, 52]. Interestingly, TAP detected this event in 21 of the Leucegene samples we analyzed. Qin et al. [53] reported that this fusion regulates cell proliferation by its transcript, not through a translated protein. Its presence in AML suggests it may be more widespread in other cancer types than previously thought.

### *FLT3*-ITD

Four *NUP98*-*NSD1*, three CBF, and two *CEBPA* patients were reported to carry a *FLT3* ITD. TAP could detect these events and additional alleles from the same samples (Additional file 2: Table S3). In addition, TAP detected *FLT3*-ITDs in 12 samples analyzed in this study (three from the CBF-cohort, five from the *MLL*-F cohort, three from the *CEBPA* cohort, and one from the *MLL*-PTD sample; see the following paragraph for a description of the latter) that were not reported previously to be positive for this event. Given the fact that the un-reported events all reside in exon 14 (same as all reported cases), and are in-frame, it is highly likely that they are true positives previously missed.

### *MLL*-PTD

Because the 23 sample accessions of the *MLL*-PTD cohort [41] were not disclosed, we analyzed about 20 samples randomly selected from a list of 377 samples that are not part of the three fusion or *CEBPA* cohorts (all negative for *MLL*-PTD), and found one positive candidate. A breakpoint suggesting a tandem duplication of exons 2 to 6, one of the most common *MLL*-PTD alleles in AML [54], was detected in sample 09H106. Although we cannot provide any precision metric due to missing information, this single positive case nonetheless highlights TAP’s ability to detect PTD events.

### *CEBPA* indels and *NPM1* insertion

TAP detected all the reported indels in 12 of the *CEBPA* samples [44]. Though, these events may be often labeled differently: for example, our pipeline may report them as duplication (06H026) or repeat-expansion events (08H065), instead of an insertion. In one *CEBPA* (08H082) and one *NUP98*-*NSD1* (11H027) samples, TAP was also able to detect a 4 bp *NPM1* insertion, an important AML biomarker, in exon 12 where most reported mutations reside [55].

### Novel splicing

Aberrant alternative splicing has been shown to be implicated in AML development [45]. We interrogated the Leucegene dataset for novel splicing events in the genes *ANPEP* (a.k.a. *CD13*), *NOTCH2*, and *FLT3*, which have been shown to express mis-spliced transcripts in AML patients [45, 46], and TAP detected the different aberrant splicing patterns identified from these studies in various samples (Table 2, Additional file 2: Table S5). Most of the events involve single or multiple exon-skipping events, with or without associated novel splice donor or acceptors. In addition to previously reported novel splicing events in these genes, TAP identifies an additional 11 different novel exon-skipping events in *FLT3* and eight different novel exon-skipping events in *NOTCH2*.

**Table 2:** Previously identified aberrant splice events [45, 46] detected in the Leucegene samples analyzed

Variant	Number of positive samples
*CD13-Va*	27
*CD13-Vc*	53
*NOTCH2-Va*	54
*NOTCH2-Vb*	77
*FLT3-Va*	92
FLT3-Vb*	51
FLT3-Vc*	3

FLT3-Vb* – skipped exon 5 and 13-bp deletion at of exon 4 3′ end instead of skipped exons 5 and 7 and partial deletions of exons 6 and 8

FLT3-Vc* – skipped exons 5,6,7 and 13-bp deletion of exon 4 at 3’end and 76-bp deletion of exon 8 at 5′ end instead of 26-bp deletion of exon 8 at 5′ end

### Computational resources and runtime

Using 580 COSMIC genes, TAP processed 100–150 million RNA-seq read pairs within 2 hours using 32 threads on a single Intel Xeon E5–2699 v3 2.30 GHz 36-core machine running CentOS 6. In comparison, TopHat-Fusion and deFuse requires around 30 and 6 hours, respectively, to process the same datasets (Additional file 1: Figure S5).

## Discussion

We developed a bioinformatics pipeline, TAP, for analyzing RNA-seq data in a targeted manner, such that anomalies of hundreds of important cancer genes can be identified within a couple of hours, making TAP highly feasible as an analysis tool in clinical genomics applications.

Using COSMIC genes as an example, we demonstrated that BBT is highly accurate and robust at classifying reads with over 99.9% accuracy. Although Bloom filters in BBT were constructed using only the reference sequences of select target genes, BBT could still extract reads containing breakpoints of structural variants. This is particularly useful in detecting gene fusions when one of the two partner genes is unforeseen and thus missing in the target set. An example of this is the promiscuous *MLL* fusions in AML. We showed in this study that TAP was able to identify the various *MLL* fusions in the *MLL*-F Leucegene cohort even when all its partners are intentionally removed from the target set.

*De novo* sequence assembly has been shown to be a useful approach for detecting structural and splicing variants on both genomic and transcriptomic datasets [25–27]. We used AML datasets to demonstrate our pipeline’s versatile ability to identify a diverse spectrum of rearrangements (gene fusions, ITDs, PTDs, indels, etc). To assess PAVFinder performance in fusion calling, we simulated sequences from a set of 448 TCGA fusions, and showed that PAVFinder outperforms two widely used methods based on an alignment-first approach in both sensitivity and specificity. Based on the benchmarking with real AML RNA-seq datasets from Leucegene, PAVFinder captured all the published events but one low coverage event, whereas all other methods evaluated have a number of events not detected. Assembly-based variant detection also offers the advantage of detection of possibly multiple breakpoint alleles of the same event with single base pair resolution. This is evident in the fusion results of all the Leucegene samples we processed (Additional file 2: Table S4), where more than one breakpoint allele of the same event can often be found within the same or among different patients. As it has been reported that different gene fusion products of the same two genes may potentially confer different oncogenic potential [56], the ability to accurately identify different gene fusion alleles could potentially be informative for diagnostics. Another example is *FLT3*-ITD, which exhibits variability in both length and position, canonically located within exon 14. We showed that PAVFinder was able to handle this variability.

Turnaround time is an important consideration in applying next-generation sequencing for clinical diagnostic applications. With the cost of sequencing rapidly decreasing, the amount of data produced is also increasing at a rate that potentially makes sequence analysis the next bottleneck in result delivery. We demonstrated that analyzing selected disease-relevant genes instead of the entire transcriptome is a viable approach, and showed that all the clinically-relevant structural variants in the target genes can be detected with 100% sensitivity, yet with a much more desirable turnaround time. A typical assembly- or alignment-first variant analysis on an entire transcriptome library of a typical sequencing depth (100 M+ reads) currently takes overnight or longer to finish. With a reduced yet comprehensive dataset (in the context of selected targets), TAP can potentially be extended to additional types of RNA-seq analysis such as SNV detection and expression profiling. Moreover, multiple tools for detecting the same kind of variants can be applied as a complementary approach, and still be feasible timewise because of a reduced data size.

## Conclusions

We developed a bioinformatics pipeline, TAP, which assembles and analyses RNA-seq data for detection structural and splicing variants. Applied on a targeted gene set, TAP shows good performance with high sensitivity and specificity with a quick turnaround time, making it a good candidate for downstream analysis on clinical sequencing.

## Availability and requirements

Project name: TAP.

Project home page: http://www.bcgsc.ca/platform/bioinfo/software/pavfinder

Operating system(s): Linux.

Programming language: Python 2.7.

Other requirements: None.

License: BCCA (academic use).

Any restrictions to use by non-academics: None.

## Additional files

Additional file 1: **Figure S1**. Read classification by Bloom-filter vs alignment. **Figure S2.** Per-gene comparison of classification performance by BBT vs BWA-MEM. **Figure S3**. Effect of sequencing error rate on performance of read classification. **Figure S4**. Support level of gene fusions detected in Leucegene samples. **Figure S5**. Benchmarking of TAP and other fusion callers. **Table S1**. Alignment features used by PAVFinder for classifying various types of transcriptomic structural variants. **Table S2**. Block-vs-exon alignment characteristics used by PAVFinder to identify various classes of novel splice variants. **Table S3**. Software and command lines used in TAP and benchmark experiments. (PDF 1076 kb)

Additional file 2: **Table S4**. AML-relevant structural variants detected by TAP in Leucegene. **Table S5**. Aberrant splicing variants previously identified in AML patients detected by TAP in Leucegene samples. (PDF 978 kb)

## Abbreviations

AML: Acute myeloid leukemia
BBT: BioBloom Tools
COSMIC: The Catalogue Of Somatic Mutations In Cancer
indel: Insertion deletion
ITD: Internal tandem duplication
PAVFinder: Post Assembly Variant Finder
PTD: Partial tandem duplication
RNA-seq: RNA sequencing
SNV: Single nucleotide variant
TAP: Targeted Assembly Pipeline
TCGA: The Cancer Genome Atlas
