# Supplementary material for: TAP: a targeted clinical genomics pipeline for detecting transcript variants using RNA-seq data
Source: BMC Med Genomics. 2018 Sep 10;11:79. doi: 10.1186/s12920-018-0402-6 (PMC6131862; doi:10.1186/s12920-018-0402-6)
Supplement: Supplementary file 1 — Figure S1. Read classification by Bloom-filter vs alignment. Figure S2. Per-gene comparison of classification performance by BBT vs BWA-MEM. Figure S3. Effect of sequencing error rate on performance of read classification. Figure S4. Support level of gene fusions detected in Leucegene samples. Figure S5. Benchmarking of TAP and other fusion callers. Table S1. Alignment features used by PAVFinder for classifying various types of transcriptomic structural variants. Table S2. Block-vs-exon alignment characteristics used by PAVFinder to identify various classes of novel splice variants. Table S3. Software and command lines used in TAP and benchmark experiments. (PDF 1076 kb) [file 12920_2018_402_MOESM1_ESM.pdf]

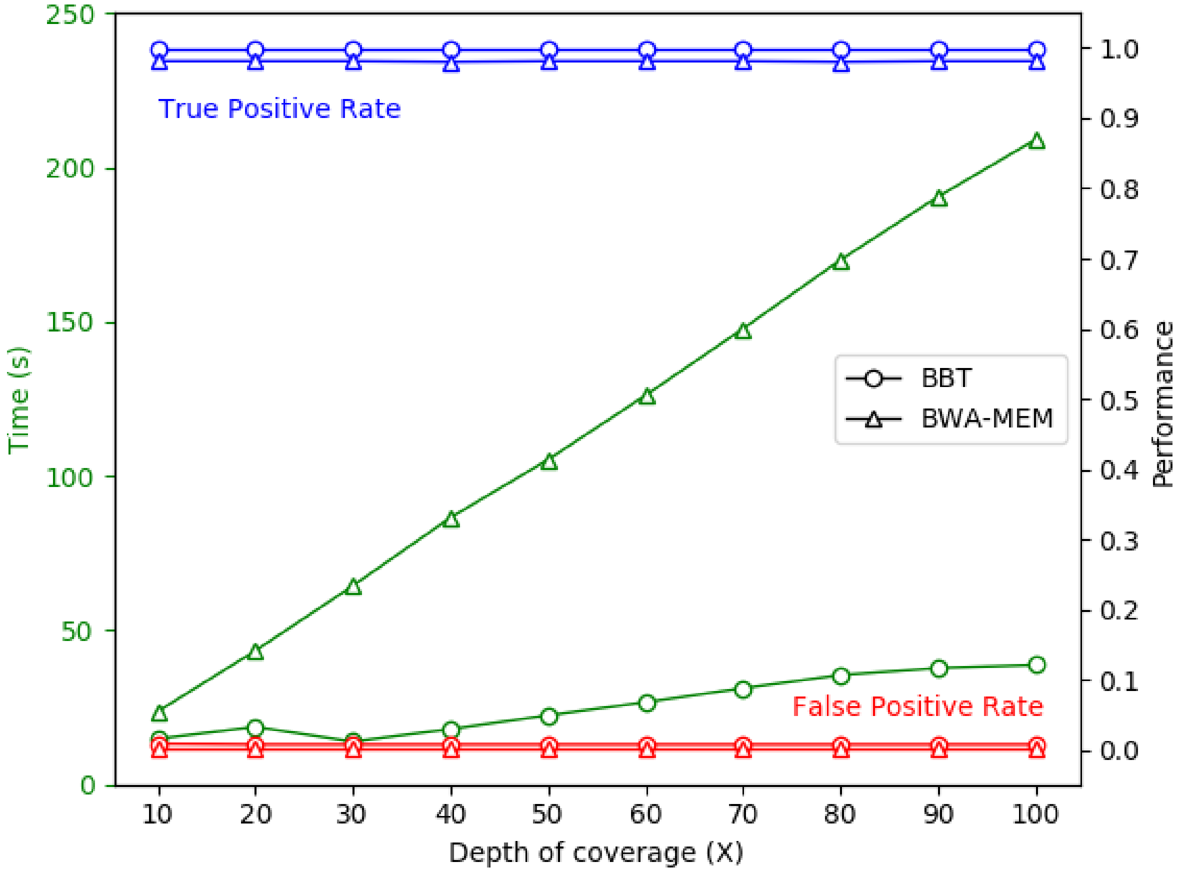

Fig. S1 Read classification by Bloom-filter vs alignment. 100bp-Illumina reads were simulated by pIRS (v1.1.1) with coverage depths ranging from 10X to 100X from 580 COSMIC (v77) genes and an equal number of non-COSMIC genes randomly selected from RefSeq. Read classification performance (right Y-axis) and run-time (green, left Y-axis) of BBT (v2.1.0) were compared against BWA-MEM (v0.7.12). The classified target of every read was compared against its true gene origin to calculate true (blue) and false (red) positive rates. Benchmarking was done in the same computational environment described in Fig. S5.

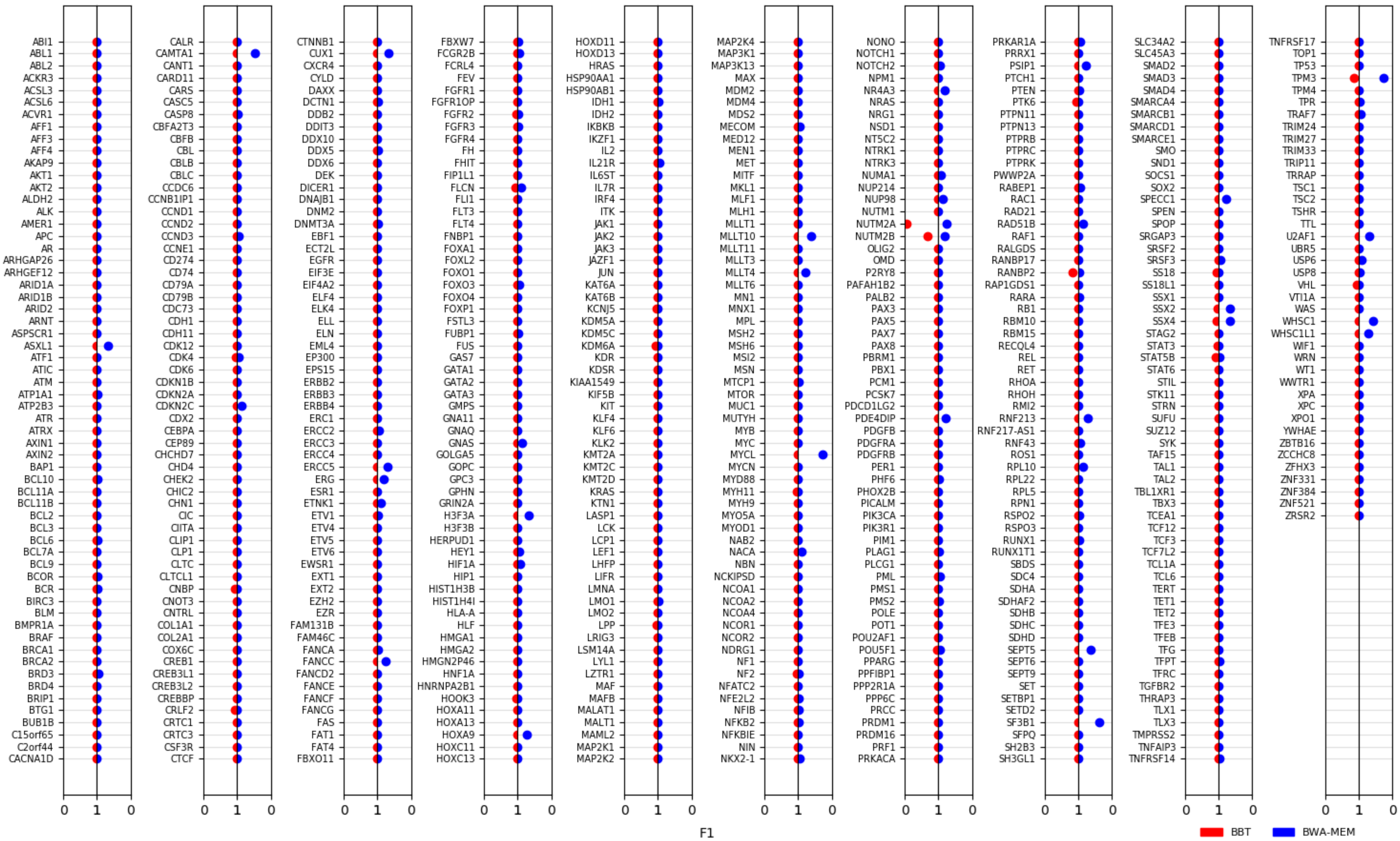
ABI1
ABL1
ABL2
ACKR3
ACSL3
ACSL6
ACVR1
AFF1
AFF3
AFF4
AKAP9
AKT1
AKT2
ALDH2
ALK
AMER1
APC
AR
ARHGAP26
ARHGEF12
ARID1A
ARID1B
ARID2
ARNT
ASPSCR1
ASXL1
ATF1
ATIC
ATM
ATP1A1
ATP2B3
ATR
ATRX
AXIN1
AXIN2
BAP1
BCL10
BCL11A
BCL11B
BCL2
BCL3
BCL6
BCL7A
BCL9
BCOR
BCR
BIRC3
BLM
BMPR1A
BRAF
BRCA1
BRCA2
BRD3
BRD4
BRIP1
BTG1
BUB1B
C15orf65
C2orf44
CACNA1D
CALR
CAMTA1
CANT1
CARD11
CARS
CASC5
CASP8
CBFA2T3
CBFB
CBL
CBLB
CBLC
CCDC6
CCNB1IP1
CCND1
CCND2
CCND3
CCNE1
CD274
CD74
CD79A
CD79B
CDC73
CDH1
CDH11
CDK12
CDK4
CDK6
CDKN1B
CDKN2A
CDKN2C
CDX2
CEBPA
CEP89
CHCHD7
CHD4
CHEK2
CHIC2
CHN1
CIC
CIITA
CLIP1
CLP1
CLTC
CLTCL1
CNBP
CNOT3
CNTRL
COL1A1
COL2A1
COX6C
CREB1
CREB3L1
CREB3L2
CREBBP
CRLF2
CRTC1
CRTC3
CSF3R
CTCF
CTNNB1
CUX1
CXCR4
CYLD
DAXX
DCTN1
DDB2
DDIT3
DDX10
DDX5
DDX6
DEK
DICER1
DNAJB1
DNM2
DNMT3A
EBF1
ECT2L
EGFR
EIF3E
EIF4A2
ELF4
ELK4
ELL
ELN
EML4
EP300
EPS15
ERBB2
ERBB3
ERBB4
ERC1
ERCC2
ERCC3
ERCC4
ERCC5
ERG
ESR1
ETNK1
ETV1
ETV4
ETV5
ETV6
EWSR1
EXT1
EXT2
EZH2
EZR
FAM131B
FAM46C
FANCA
FANCC
FANCD2
FANCE
FANCF
FANCG
FAS
FAT1
FAT4
FBXO11
FBXW7
FCGR2B
FCRL4
FEV
FGFR1
FGFR1OP
FGFR2
FGFR3
FGFR4
FH
FHIT
FIP1L1
FLCN
FLI1
FLT3
FLT4
FNBP1
FOXA1
FOXL2
FOXO1
FOXO3
FOXO4
FOXP1
FSTL3
FUBP1
FUS
GAS7
GATA1
GATA2
GATA3
GMPS
GNA11
GNAQ
GNAS
GOLGA5
GOPC
GPC3
GPHN
GRIN2A
H3F3A
H3F3B
HERPUD1
HEY1
HIF1A
HIP1
HIST1H3B
HIST1H4I
HLA-A
HLF
HMGA1
HMGA2
HMGN2P46
HNF1A
HNRNPA2B1
HOOK3
HOXA11
HOXA13
HOXA9
HOXC11
HOXC13
HOXD11
HOXD13
HRAS
HSP90AA1
HSP90AB1
IDH1
IDH2
IKBKB
IKZF1
IL2
IL21R
IL6ST
IL7R
IRF4
ITK
JAK1
JAK2
JAK3
JAZF1
JUN
KAT6A
KAT6B
KCNJ5
KDM5A
KDM5C
KDM6A
KDR
KDSR
KIAA1549
KIF5B
KIT
KLF4
KLF6
KLK2
KMT2A
KMT2C
KMT2D
KRAS
KTN1
LASP1
LCK
LCP1
LEF1
LHFP
LIFR
LMNA
LMO1
LMO2
LPP
LRIG3
LSM14A
LYL1
LZTR1
MAF
MAFB
MALAT1
MALT1
MAML2
MAP2K1
MAP2K2
MAP2K4
MAP3K1
MAP3K13
MAX
MDM2
MDM4
MDS2
MECOM
MED12
MEN1
MET
MITF
MKL1
MLF1
MLH1
MLLT1
MLLT10
MLLT11
MLLT3
MLLT4
MLLT6
MN1
MNX1
MPL
MSH2
MSH6
MSI2
MTCP1
MTOR
MUC1
MUTYH
MYB
MYC
MYCL
MYCN
MYD88
MYH11
MYH9
MYO5A
MYOD1
NAB2
NACA
NBN
NCKIPSD
NCOA1
NCOA2
NCOA4
NCOR1
NCOR2
NDRG1
NF1
NF2
NFATC2
NFE2L2
NFIB
NFKB2
NFKBIE
NIN
NKX2-1
NONO
NOTCH1
NOTCH2
NPM1
NR4A3
NRAS
NRG1
NSD1
NT5C2
NTRK1
NTRK3
NUMA1
NUP214
NUP98
NUTM1
NUTM2A
NUTM2B
OLIG2
OMD
P2RY8
PAFAH1B2
PALB2
PAX3
PAX5
PAX7
PAX8
PBRM1
PBX1
PCM1
PCSK7
PDCD1LG2
PDE4DIP
PDGFB
PDGFRA
PDGFRB
PER1
PHF6
PHOX2B
PICALM
PIK3CA
PIK3R1
PIM1
PLAG1
PLCG1
PML
PMS1
PMS2
POLE
POT1
POU2AF1
POU5F1
PPARG
PPFIBP1
PPP2R1A
PPP6C
PRCC
PRDM1
PRDM16
PRF1
PRKACA
PRKAR1A
PRRX1
PSIP1
PTCH1
PTEN
PTK6
PTPN11
PTPN13
PTPRB
PTPRC
PTPRK
PWWP2A
RABEP1
RAC1
RAD21
RAD51B
RAF1
RALGDS
RANBP17
RANBP2
RAP1GDS1
RARA
RB1
RBM10
RBM15
RECQL4
REL
RET
RHOA
RHOH
RMI2
RNF213
RNF217-AS1
RNF43
ROS1
RPL10
RPL22
RPL5
RPN1
RSPO2
RSPO3
RUNX1
RUNX1T1
SBDS
SDC4
SDHA
SDHAF2
SDHB
SDHC
SDHD
SEPT5
SEPT6
SEPT9
SET
SETBP1
SETD2
SF3B1
SFPQ
SH2B3
SH3GL1
SLC34A2
SLC45A3
SMAD2
SMAD3
SMAD4
SMARCA4
SMARCB1
SMARCD1
SMARCE1
SMO
SND1
SOCS1
SOX2
SPECC1
SPEN
SPOP
SRGAP3
SRSF2
SRSF3
SS18
SS18L1
SSX1
SSX2
SSX4
STAG2
STAT3
STAT5B
STAT6
STIL
STK11
STRN
SUFU
SUZ12
SYK
TAF15
TAL1
TAL2
TBL1XR1
TBX3
TCEA1
TCF12
TCF3
TCF7L2
TCL1A
TCL6
TERT
TET1
TET2
TFE3
TFEB
TFG
TFPT
TFRC
TGFBR2
THRAP3
TLX1
TLX3
TMPRSS2
TNFAIP3
TNFRSF14
TNFRSF17
TOP1
TP53
TPM3
TPM4
TPR
TRAF7
TRIM24
TRIM27
TRIM33
TRIP11
TRRAP
TSC1
TSC2
TSHR
TTL
U2AF1
UBR5
USP6
USP8
VHL
VTI1A
WAS
WHSC1
WHSC1L1
WIF1
WRN
WT1
WWTR1
XPA
XPC
XPO1
YWHAE
ZBTB16
ZCCHC8
ZFHX3
ZNF331
ZNF384
ZNF521
ZRSR2
0
1
0
F1
BBT
BWA-MEM

Fig. S2 Per-gene comparison of classification performance by BBT vs BWA-MEM. F1 scores for both methods using the same simulation dataset described in Fig. S1 is calculated for each gene and plotted on the same horizontal line (red=BBT, blue=BWA-mem). The scale on the X-axis for BWA-mem on the right is reversed for easy visual comparison such that higher scores for both methods localize to the middle while lower scores are off-centre. F1 score is calculated as follows: F1 = 2pr/(p + r); where p(precision) = number of correctly assigned reads / total reads assigned; r(recall) = number of correctly assigned reads / total reads simulated (for the gene in question).

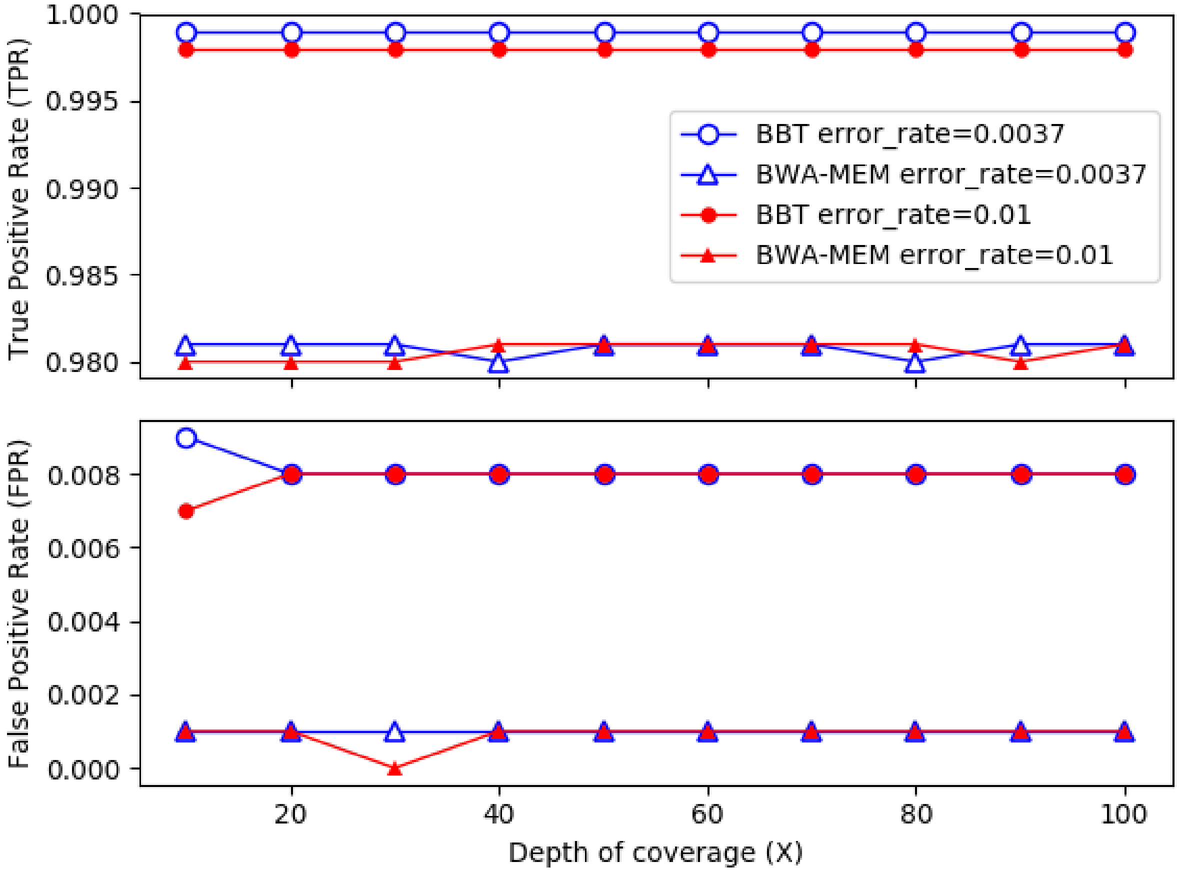

Fig. S3 Effect of sequencing error rate on performance of read classification. Simulation was repeated with increased substitution-error rate of 1% (red and solid data points) and results were compared against the original experiment (Figure S1, 0.37% substitution-error rate, blue and hollow data points). True (top panel) and false positive rates (bottom panel) of both methods, BBT(circle) and BWA-mem(triangle), were plotted against depth of coverage (X-axis).

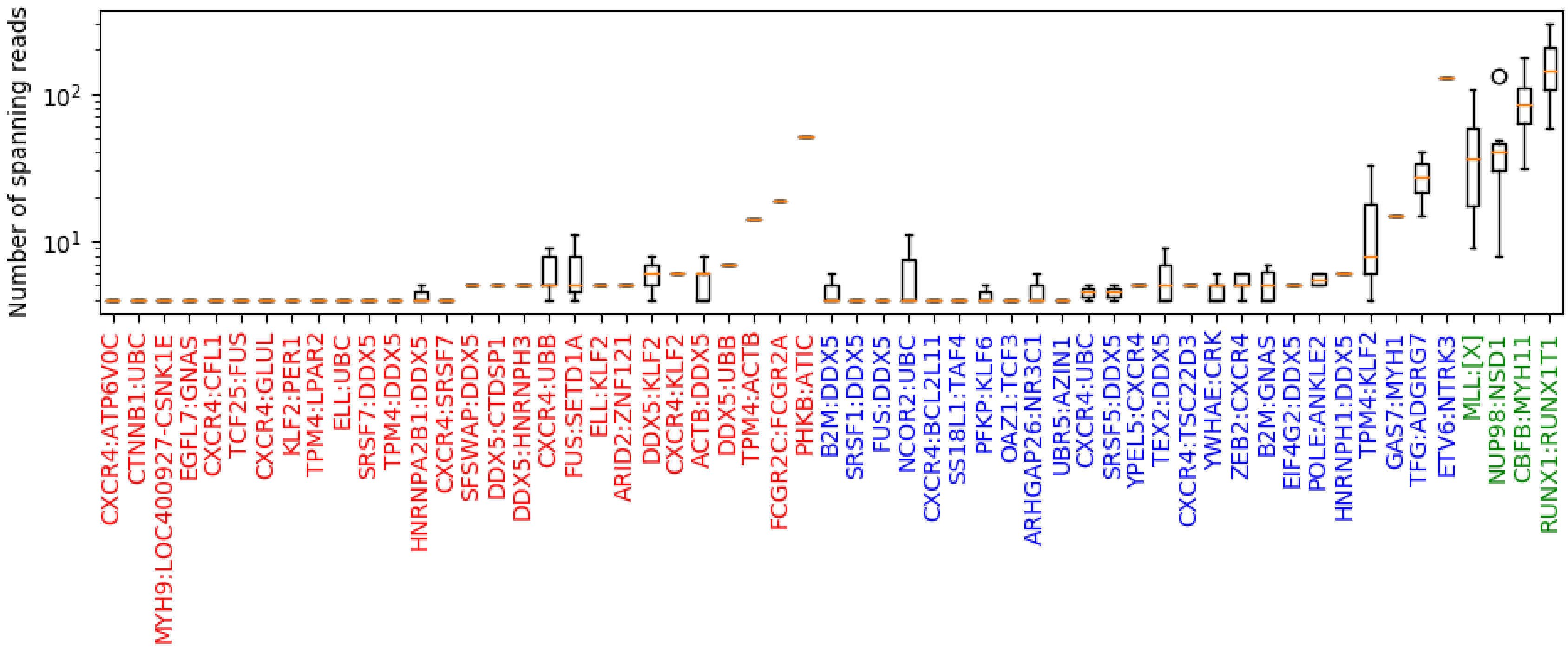

Fig. S4 Support level of gene fusions detected in Leucegene samples. A boxplot is shown to indicate the distribution of the number of junction spanning reads (Y-axis, log-scale) of each fusion event detected in Leucegene samples analyzed in this study (X-axis, grouped by gene names regardless of orientation; *MLL* fusions with different partners in the *MLL*-F cohort were grouped as MLL:[X]). Events were colored based on their level of “legitimacy”: green = AML target events (events targeted in Leucegene publications); blue = non-target events with literature support; red = non-target events without literature support. List of non-target events with their associated literature support: B2M:DDX5[1], SRSF5:DDX5[1], FUS:DDX5[1], NCOR2:UBC[2], CXCR4:BCL2L11[1], SS18L1:TAF4[3], PFKP:KLF6[1], OAZ1:TCF3[1], ARHGAP26:NR3C1[1], UBR5:AZIN1[4], CXCR4:UBC[1], SRSF1:DDX5[1], YPEL5:CXCR4[1], TEX2:DDX5[1], CXCR4:TSC22D3[1], YWHAE:CRK[1], ZEB2:CXCR4[1], B2M:GNAS[1], EIF4G2:DDX5[1], POLE:ANKLE2[1], HNRNPH1:DDX5[1], TPM4:KLF2[5], GAS7:MYH1[6], TFG:ADGRG7[7], ETV6:NTRK3[8]

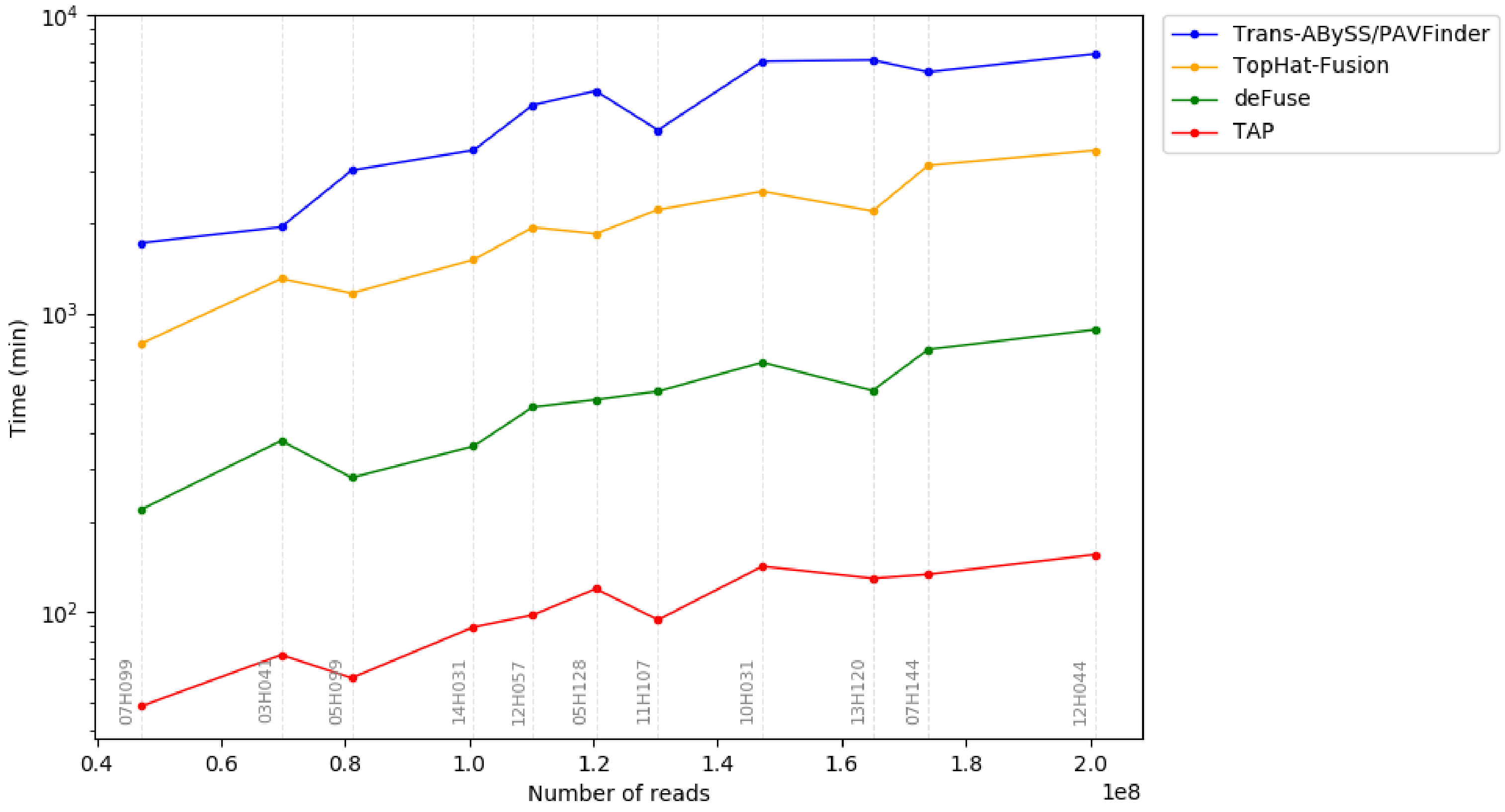

Fig. S5 Benchmarking of TAP and other fusion callers. TAP, Trans-ABySS/PAVFinder, TopHat-Fusion (v2.1.0), and deFuse (v0.8.0) were run on eleven Leucegene samples with sequencing depths ranging from 50 to 200 million read pairs. Target gene set for TAP comprises 580 COSMIC (v77) genes; the other 3 tools /pipelines were run on entire transcriptome. TopHat-Fusion and deFuse were run with default parameters and references as per their instructions. The second step of TopHat-Fusion (tophat-fusion-post) failed to finish on these samples as it requires in excess of 380GB which is the upper limit of memory available on the testing machines. All benchmarking were performed on Dell C6320 Intel E5 2.2Ghz 48-core 2.2Ghz machines with 384GB RAM running CentOS 6.7.

Table S1 Alignment features used by PAVFinder for classifying various types of transcriptomic structural variants. Types of alignments, alignment strands relative to both the genome and transcript, gene loci involved are used to elucidate the underlying rearrangement each contig with aberrant alignment reconstructs.

Event type	c2g alignment type	Gene loci mapped	c2g strands	Gene strands	Breakpoints flush with exon boundaries
gene fusion	Split	2	NA	same	yes*
read-through	Split/Gapped	2	same	same	yes
internal tandem duplication (ITD)	Split/Gapped/Partial**	1	same	same	NA
partial tandem duplication (PTD)	Split	1	same	same	yes
small insertion	Gapped	1	NA	same	NA
small deletion	Gapped	1	NA	same	NA

c2g strands: reference genome strand contig sequence is aligned to
Gene strands: strand of c2g alignment relative to gene strand
Split alignment: contig is split into 2 segments aligned to non-adjacent locations of the genome (chimeric alignment)
Gapped alignment: contig is aligned to a single genomic but with gap(s) either in the contig or target (genome) sequence not corresponding to introns
NA: not applicable
* Default requirement for fusion calling; parameter can be turned off to allow non-exon-bound fusions to be called
** Contig harboring ITD breakpoints may lead to partial c2g alignments, in which case the unaligned portion will be aligned against the target transcript (determined by c2g) sequence for prediction of an ITD event

Table S2 Block-vs-exon alignment characteristics used by PAVFinder to identify various classes of novel splice variants. Alignment of an assembled transcritptomic contig against the reference genome (c2g) yields alignment “blocks”, with each block corresponding to an exon when a contig reconstructs a reference transcript. When novel splicing not observed in the annotation happens, the colinearity of blocks-vs-exons is disrupted, yielding clues for classifying different types of splice variants.

Event	Block-vs-exon alignment
skipped exon	adjacent contig blocks mapped to non-adjacent exons
novel exon	adjacent exons mapped to non-adjacent contig blocks
novel intron	adjacent contig blocks mapped to single exon with outer boundaries flush
retained intron	adjacent exons mapped to single block with outer boundaries flush
novel splice donor	novel block boundary corresponding to the splice donor of the matching exon
novel splice acceptor	novel block boundary corresponding to the splice acceptor of the matching exon

All the splice variants require the novel block(s) to be flanked by the canonical splicing motif (GT-AG).

Table S3 Software and command lines used in TAP and benchmark experiments

software	version	command
**pIRS**	1.1.1	pirs simulate -i <reference.fa> -s </pIRS_1111/Profiles/Base-Calling_Profiles/humNew.PE100.matrix.gz> -b </pIRS_1111/Profiles/InDel_Profiles/phixv2.InDel.matrix> -d </pIRS_1111/Profiles/GC-depth_Profiles/humNew.gcdep_100.dat> -x <coverage_depth> -m 150 -Q 33 -c 1 -o <output_prefix> [-e <error_rate>]
**biobloommaker**	2.1.0	biobloommaker -F -f 0.005 -p cancer_census -a 4 -m -S "0000010011001010100100110000001011011101011000101110100110111111101010000111011000000001010101110010100000010000000000000010011100110001100011000100100100011000110001100111001000000000000010000001010011101010100000000110111000010101111111011001011101000110101110110100000011001001010100110010000" cancer_census/*.fa
**TAP**	0.4.2	tap.py <output_prefix> <output_directory> --bf </path/biobloommaker/output.bf> --fq </path/xxx_1.fastq.gz> </path/xxx_2.fastq.gz> --k 32 62 --readlen <read_length> --nprocs <number_processes> --params </path/to/tap.cfg>
**biobloomcategorizer**	2.1.0	biobloomcategorizer -fq -i -p <output_prefix> -a 2 -t <num_threads> -e -f </path/biobloommaker/output.bf> </path/xxx_1.fastq> </path/xxx_2.fastq>
**Trans-ABySS**	1.5.4	transabyss --kmer <kmer_size> --pe </path/xxx_1.fastq.gz> </path/xxx_2.fastq.gz> -outdir <output_directory> --name <prefix_name> --cleanup 3<br>transabyss-merge -mink 32 -maxk 62 --prefixes k32 k62 -length 100 %s --out <output_file> --force
**GMAP**	2014-12-18	gmap -D </path/gmap_index/> -d hg19 <input.fasta> -t <num_threads> -f samse -n 0 -x 10
**PAVFinder**	0.4.2	find_sv_transcriptome.py --gbam </path/c2g.bam> --tbam </path/c2t.bam> --transcripts_fasta </path/transcripts.fasta> --genome_index </path/gmap_index/> --r2c </path/r2c.bam> --nproc <number_process> </path/contigs.fasta> </path/refGene.sorted.gtf.gz> </path/hg19.fa> <output_dir><br>map_splice.py </path/c2g.bam> </path/contigs.fasta> </path/refGene.sorted.gtf.gz> </path/hg19.fa> <output_dir> --r2c </path/r2c.bam> --nproc <number_process> --suppl_annot

<table>
<tr><th></th><th></th><th></th></tr>
<tr><td></td><td></td><td></path/acembly.sorted.gtf.gz></td></tr>
<tr><td>BWA MEM</td><td>0.7.12</td><td>bwa mem -t <number_threads> </path/reference/bwa/index> </path/xxx_1.fastq.gz> </path/xxx_2.fastq.gz> | samtools view -bhS - -o <output.bam></td></tr>
<tr><td>TopHat-Fusion</td><td>2.1.0</td><td>tophat –o <output_directory> -p 12 --fusion-search --keep-fasta-order --bowtie1 --no-coverage-search -r 0 --mate-std-dev 80 --max-intron-length 100000 --fusion-min-dist 100000 --fusion-anchor-length 13 --fusion-ignore-chromosomes chrM </path/bowtie/hg19/index/> </path/xxx_1.fastq> </path/xxx_2.fastq></td></tr>
<tr><td>deFuse</td><td>0.6.1</td><td>defuse_run.pl –c </path/config.txt> -d </path/to/defuse_ref/> -o <output_directory> -1 </path/xxx_1.fastq> -2 </path/xxx_2.fastq> –n <name> -p 8</td></tr>
</table>

Reference:

1. Marincevic-Zuniga Y, Dahlberg J, Nilsson S, Raine A, Nystedt S, Lindqvist CM, Berglund EC, Abrahamsson J, Cavelier L, Forestier E *et al*: **Transcriptome sequencing in pediatric acute lymphoblastic leukemia identifies fusion genes associated with distinct DNA methylation profiles**. *Journal of hematology & oncology* 2017, **10**(1):148.
2. Li Y, Heavican TB, Vellichirammal NN, Iqbal J, Guda C: **ChimeRScope: a novel alignment-free algorithm for fusion transcript prediction using paired-end RNA-Seq data**. *Nucleic acids research* 2017, **45**(13):e120.
3. **Atlas of Genetics and Cytogenetics in Oncology and Haematology** [http://AtlasGeneticsOncology.org]
4. Ruffle F AJ, Boureux A et al.: **New chimeric RNAs in acute myeloid leukemia [version 1; referees: 1 approved]**. *F1000Research* 2017, **6(ISCB Comm J)**:1302.
5. Roberts KG, Morin RD, Zhang J, Hirst M, Zhao Y, Su X, Chen SC, Payne-Turner D, Churchman ML, Harvey RC *et al*: **Genetic alterations activating kinase and cytokine receptor signaling in high-risk acute lymphoblastic leukemia**. *Cancer cell* 2012, **22**(2):153-166.
6. Yoshihara K, Wang Q, Torres-Garcia W, Zheng S, Vegesna R, Kim H, Verhaak RG: **The landscape and therapeutic relevance of cancer-associated transcript fusions**. *Oncogene* 2015, **34**(37):4845-4854.
7. Chase A, Ernst T, Fiebig A, Collins A, Grand F, Erben P, Reiter A, Schreiber S, Cross NC: **TFG, a target of chromosome translocations in lymphoma and soft tissue tumors, fuses to GPR128 in healthy individuals**. *Haematologica* 2010, **95**(1):20-26.
8. Lannon CL, Sorensen PH: **ETV6-NTRK3: a chimeric protein tyrosine kinase with transformation activity in multiple cell lineages**. *Seminars in cancer biology* 2005, **15**(3):215-223.
