# Supplementary material for: TAP: a targeted clinical genomics pipeline for detecting transcript variants using RNA-seq data
Source: BMC Med Genomics. 2018 Sep 10;11:79. doi: 10.1186/s12920-018-0402-6 (PMC6131862; doi:10.1186/s12920-018-0402-6)
Supplement: Supplementary file 2 — Table S4. AML-relevant structural variants detected by TAP in Leucegene. Table S5. Aberrant splicing variants previously identified in AML patients detected by TAP in Leucegene samples. (PDF 978 kb) [file 12920_2018_402_MOESM2_ESM.pdf]

Table S4. AML-relevant structural variants detected by TAP in Leucegene samples

sample	event	coord1	coord2	5'gene	3'gene	5'exon	3'exon	in_frame	size	spanning_reads
03H065	fusion	chr8:93029591	chr21:36231770	RUNX1	RUNX1T1	6	6	T	na	108
03H065	fusion	chr8:93074855	chr21:36231770	RUNX1	RUNX1T1	6	5	F	na	7
03H065	fusion	chr8:93074937	chr21:36231770	RUNX1	RUNX1T1	6	2	F	na	13
03H083	fusion	chr8:93029591	chr21:36231770	RUNX1	RUNX1T1	6	6	T	na	108
03H095	fusion	chr16:15818197	chr16:67116210	CBFB	MYH11	5	32	T	na	93
03H095	fusion	chr16:15818198	chr16:67116209	CBFB	MYH11	5	32	T	na	32
03H109	fusion	chr16:15814908	chr16:67116241	CBFB	MYH11	5	34	F	na	7
03H112	fusion	chr16:15814908	chr16:67116210	CBFB	MYH11	5	34	T	na	75
03H112	ITD	chr13:28608084	chr13:28608263	FLT3	FLT3	15	14	T	93	115
04H030	fusion	chr16:15814908	chr16:67116210	CBFB	MYH11	5	34	T	na	80
04H030	ITD	chr13:28608253	chr13:28608309	FLT3	FLT3	14	14	T	57	17
04H061	fusion	chr16:15814908	chr16:67116210	CBFB	MYH11	5	34	T	na	41
04H091	fusion	chr16:15820911	chr16:67116210	CBFB	MYH11	5	29	T	na	82
05H042	fusion	chr8:93029591	chr21:36231770	RUNX1	RUNX1T1	6	6	T	na	182
05H042	fusion	chr8:93074937	chr21:36231770	RUNX1	RUNX1T1	6	2	F	na	123
05H099	fusion	chr16:15814908	chr16:67116210	CBFB	MYH11	5	34	T	na	48
05H113	fusion	chr16:15814908	chr16:67116210	CBFB	MYH11	5	34	T	na	88
05H118	fusion	chr8:93029591	chr21:36231770	RUNX1	RUNX1T1	6	6	T	na	117
05H118	fusion	chr8:93074855	chr21:36231770	RUNX1	RUNX1T1	6	5	F	na	15
05H118	fusion	chr8:93074937	chr21:36231770	RUNX1	RUNX1T1	6	2	F	na	52
05H136	fusion	chr16:15814908	chr16:67116210	CBFB	MYH11	5	34	T	na	86
05H136	fusion	chr16:15814908	chr16:67116241	CBFB	MYH11	5	34	F	na	4
05H184	fusion	chr8:93023319	chr21:36231770	RUNX1	RUNX1T1	6	8	F	na	13
05H184	fusion	chr8:93029591	chr21:36231770	RUNX1	RUNX1T1	6	6	T	na	277
05H184	fusion	chr8:93074855	chr21:36231770	RUNX1	RUNX1T1	6	5	F	na	19
05H184	fusion	chr8:93074937	chr21:36231770	RUNX1	RUNX1T1	6	2	F	na	103
06H020	fusion	chr16:15814908	chr16:67116210	CBFB	MYH11	5	34	T	na	117
06H020	fusion	chr16:15814908	chr16:67116241	CBFB	MYH11	5	34	F	na	6
06H035	fusion	chr8:93029591	chr21:36231770	RUNX1	RUNX1T1	6	6	T	na	166
06H035	fusion	chr8:93074937	chr21:36231770	RUNX1	RUNX1T1	6	2	F	na	19
06H115	fusion	chr16:15814908	chr16:67116210	CBFB	MYH11	5	34	T	na	109

06H115	fusion	chr16:15814908	chr16:67116241	CBFB	MYH11	5	34	F	na	11
07H099	fusion	chr16:15814908	chr16:67116210	CBFB	MYH11	5	34	T	na	44
07H137	fusion	chr8:93029591	chr21:36231770	RUNX1	RUNX1T1	6	6	T	na	255
07H144	fusion	chr16:15814908	chr16:67116210	CBFB	MYH11	5	34	T	na	158
07H144	fusion	chr16:15814908	chr16:67116241	CBFB	MYH11	5	34	F	na	10
08H034	fusion	chr8:93029591	chr21:36231770	RUNX1	RUNX1T1	6	6	T	na	99
08H034	fusion	chr8:93074855	chr21:36231770	RUNX1	RUNX1T1	6	5	F	na	4
08H034	fusion	chr8:93074937	chr21:36231770	RUNX1	RUNX1T1	6	2	F	na	47
08H042	fusion	chr8:93029591	chr21:36231770	RUNX1	RUNX1T1	6	6	T	na	140
08H042	fusion	chr8:93074937	chr21:36231770	RUNX1	RUNX1T1	6	2	F	na	36
08H072	fusion	chr8:93029591	chr21:36231770	RUNX1	RUNX1T1	6	6	T	na	252
08H072	fusion	chr8:93074855	chr21:36231770	RUNX1	RUNX1T1	6	5	F	na	23
08H072	fusion	chr8:93074937	chr21:36231770	RUNX1	RUNX1T1	6	2	F	na	103
08H072	ins	chr13:28608248	chr13:28608248	FLT3	FLT3	14	14	T	21	98
08H081	fusion	chr16:15814908	chr16:67116210	CBFB	MYH11	5	34	T	na	135
08H081	fusion	chr16:15814908	chr16:67116241	CBFB	MYH11	5	34	F	na	30
08H099	fusion	chr16:15814908	chr16:67116210	CBFB	MYH11	5	34	T	na	65
08H099	fusion	chr16:15814908	chr16:67116241	CBFB	MYH11	5	34	F	na	8
09H016	fusion	chr16:15814908	chr16:67116210	CBFB	MYH11	5	34	T	na	79
09H040	fusion	chr8:93029591	chr21:36231770	RUNX1	RUNX1T1	6	6	T	na	299
09H040	fusion	chr8:93074937	chr21:36231770	RUNX1	RUNX1T1	6	2	F	na	79
09H066	fusion	chr16:15814908	chr16:67116210	CBFB	MYH11	5	34	T	na	31
10H008	fusion	chr16:15818849	chr16:67116210	CBFB	MYH11	5	30	T	na	129
10H008	fusion	chr16:15818849	chr16:67116241	CBFB	MYH11	5	30	F	na	12
10H030	fusion	chr8:93029591	chr21:36231770	RUNX1	RUNX1T1	6	6	T	na	127
10H030	fusion	chr8:93074855	chr21:36231770	RUNX1	RUNX1T1	6	5	F	na	4
10H030	fusion	chr8:93074937	chr21:36231770	RUNX1	RUNX1T1	6	2	F	na	33
10H119	fusion	chr8:93029591	chr21:36231770	RUNX1	RUNX1T1	6	6	T	na	219
11H022	fusion	chr16:15814908	chr16:67116210	CBFB	MYH11	5	34	T	na	41
11H022	fusion	chr16:15814908	chr16:67116241	CBFB	MYH11	5	34	F	na	8
11H022	ins	chr13:28608269	chr13:28608269	FLT3	FLT3	14	14	T	3	53
11H104	fusion	chr16:15814908	chr16:67116210	CBFB	MYH11	5	34	T	na	71
11H107	fusion	chr8:93029591	chr21:36231770	RUNX1	RUNX1T1	6	6	T	na	203
11H107	fusion	chr8:93074855	chr21:36231770	RUNX1	RUNX1T1	6	5	F	na	4

11H107	fusion	chr8:93074937	chr21:36231770	RUNX1	RUNX1T1	6	2	F	na	22
11H179	fusion	chr16:15814908	chr16:67116210	CBFB	MYH11	5	34	T	na	62
12H042	fusion	chr16:15814920	chr16:67116210	CBFB	MYH11	5	None	F	na	45
12H044	fusion	chr16:15814908	chr16:67116210	CBFB	MYH11	5	34	T	na	112
12H045	fusion	chr8:93029591	chr21:36231770	RUNX1	RUNX1T1	6	6	T	na	188
12H098	fusion	chr8:93029591	chr21:36231770	RUNX1	RUNX1T1	6	6	T	na	77
12H098	fusion	chr8:93074937	chr21:36231770	RUNX1	RUNX1T1	6	2	F	na	38
12H165	fusion	chr16:15814169	chr16:67116210	CBFB	MYH11	5	35	T	na	32
12H165	fusion	chr16:15814908	chr16:67116210	CBFB	MYH11	5	34	T	na	86
12H166	fusion	chr8:93029591	chr21:36231770	RUNX1	RUNX1T1	6	6	T	na	112
12H166	fusion	chr8:93074855	chr21:36231770	RUNX1	RUNX1T1	6	5	F	na	7
12H166	fusion	chr8:93074937	chr21:36231770	RUNX1	RUNX1T1	6	2	F	na	24
12H180	fusion	chr8:93029591	chr21:36231770	RUNX1	RUNX1T1	6	6	T	na	149
12H180	fusion	chr8:93074937	chr21:36231770	RUNX1	RUNX1T1	6	2	F	na	29
12H180	ITD	chr13:28608273	chr13:28608334	FLT3	FLT3	14	14	T	63	38
12H183	fusion	chr8:93029591	chr21:36231770	RUNX1	RUNX1T1	6	6	T	na	59
12H183	fusion	chr8:93074937	chr21:36231770	RUNX1	RUNX1T1	6	2	F	na	19
13H066	fusion	chr16:15814908	chr16:67116210	CBFB	MYH11	5	34	T	na	107
13H066	fusion	chr16:15814908	chr16:67116241	CBFB	MYH11	5	34	F	na	13
13H120	fusion	chr16:15814908	chr16:67116210	CBFB	MYH11	5	34	T	na	175
13H120	ITD	chr13:28608219	chr13:28608331	FLT3	FLT3	14	14	F	114	36
13H169	fusion	chr8:93029591	chr21:36231770	RUNX1	RUNX1T1	6	6	T	na	72
13H169	fusion	chr8:93074855	chr21:36231770	RUNX1	RUNX1T1	6	5	F	na	9
13H169	fusion	chr8:93074937	chr21:36231770	RUNX1	RUNX1T1	6	2	F	na	39
01H001	fusion	chr6:168265231	chr11:118353209	KMT2A	MLLT4	8	2	T	na	40
02H017	fusion	chr11:118355029	chr19:6218051	KMT2A	MLLT1	9	7	T	na	28
02H032	fusion	chr11:118353210	chr17:36868099	KMT2A	MLLT6	8	7	T	na	9
03H067	fusion	chr11:118352807	chr17:75303222	KMT2A	SEPT9	7	2	T	na	54
04H041	fusion	chr11:118352807	chr17:75303222	KMT2A	SEPT9	7	2	T	na	31
04H121	fusion	chr11:118355029	chr19:18583691	KMT2A	ELL	9	2	T	na	37
04H121	fusion	chr11:118355690	chr19:18583691	KMT2A	ELL	10	2	T	na	24
04H121	fusion	chr11:118359329	chr19:18632730	ELL	KMT2A	1	11	T	na	8
04H121	ITD	chr13:28608262	chr13:28608341	FLT3	FLT3	14	14	T	75	9
05H025	fusion	chr9:20365742	chr11:118353209	KMT2A	MLLT3	8	6	T	na	23

05H066	fusion	chr6:168265231	chr11:118352806	KMT2A	MLLT4	7	2	F	na	9
05H066	fusion	chr6:168265231	chr11:118353209	KMT2A	MLLT4	8	2	T	na	85
05H066	ITD	chr13:28608270	chr13:28608307	FLT3	FLT3	14	14	T	39	14
05H128	fusion	chr10:21959378	chr11:118353209	KMT2A	MLLT10	8	10	T	na	47
06H066	fusion	chr11:118353210	chr15:40920267	KMT2A	CASC5	8	12	T	na	61
06H088	fusion	chr6:168265231	chr11:118353209	KMT2A	MLLT4	8	2	T	na	60
06H117	fusion	chr6:168265231	chr11:118353209	KMT2A	MLLT4	8	2	T	na	107
06H152	fusion	chr11:118352807	chr17:9923213	KMT2A	GAS7	7	2	F	na	9
06H152	fusion	chr11:118353210	chr17:9923213	KMT2A	GAS7	8	2	T	na	36
06H152	fusion	chr11:118354898	chr17:10101524	GAS7	KMT2A	1	9	T	na	19
06H152	ITD	chr13:28608262	chr13:28608341	FLT3	FLT3	14	14	T	75	5
07H003	fusion	chr11:118355029	chr19:18583691	KMT2A	ELL	9	2	T	na	35
07H003	fusion	chr11:118355690	chr19:18583691	KMT2A	ELL	10	2	T	na	4
07H003	fusion	chr11:118359329	chr19:18632730	ELL	KMT2A	1	11	T	na	10
07H003	ITD	chr13:28608252	chr13:28608277	FLT3	FLT3	14	14	T	27	54
07H041	fusion	chr9:20365742	chr11:118353209	KMT2A	MLLT3	8	6	T	na	39
07H045	fusion	chr11:118353210	chr19:6222694	KMT2A	MLLT1	8	6	T	na	17
07H045	ITD	chr13:28608233	chr13:28608350	FLT3	FLT3	14	14	F	119	6
07H160	fusion	chr10:21940602	chr11:118353209	KMT2A	MLLT10	8	9	T	na	11
08H021	fusion	chr9:20365742	chr11:118355028	KMT2A	MLLT3	9	6	T	na	14
08H021	fusion	chr9:20365742	chr11:118355689	KMT2A	MLLT3	10	6	T	na	7
08H085	fusion	chr11:118355029	chr19:6270769	KMT2A	MLLT1	9	2	T	na	7
08H085	fusion	chr11:118355690	chr19:6270769	KMT2A	MLLT1	10	2	T	na	12
08H129	fusion	chr6:168265231	chr11:118352806	KMT2A	MLLT4	7	2	F	na	5
08H129	fusion	chr6:168265231	chr11:118353209	KMT2A	MLLT4	8	2	T	na	68
08H139	fusion	chr11:118355029	chr19:6270769	KMT2A	MLLT1	9	2	T	na	16
09H010	fusion	chr9:20353594	chr11:118353209	KMT2A	MLLT3	8	10	T	na	10
09H018	fusion	chr11:118353210	chr19:18576727	KMT2A	ELL	8	3	T	na	40
09H018	fusion	chr11:118354898	chr19:18583644	ELL	KMT2A	2	9	T	na	23
09H032	fusion	chr9:20354877	chr11:118353209	KMT2A	MLLT3	8	9	T	na	18
09H098	fusion	chr9:20365742	chr11:118355028	KMT2A	MLLT3	9	6	T	na	19
10H031	fusion	chr6:168265231	chr11:118352806	KMT2A	MLLT4	7	2	F	na	6
10H031	fusion	chr6:168265231	chr11:118353209	KMT2A	MLLT4	8	2	T	na	73
10H058	fusion	chr9:20365742	chr11:118353209	KMT2A	MLLT3	8	6	T	na	11

10H127	fusion	chr9:20365742	chr11:118353209	KMT2A	MLLT3	8	6	T	na	41
10H127	ITD	chr13:28608262	chr13:28608341	FLT3	FLT3	14	14	T	75	4
12H057	fusion	chr6:168265231	chr11:118352806	KMT2A	MLLT4	7	2	F	na	12
12H057	fusion	chr6:168265231	chr11:118353209	KMT2A	MLLT4	8	2	T	na	76
14H031	fusion	chr6:168265231	chr11:118353209	KMT2A	MLLT4	8	2	T	na	61
03H041	fusion	chr5:176662822	chr11:3765738	NUP98	NSD1	12	7	T	na	8
03H041	ins	chr13:28608247	chr13:28608247	FLT3	FLT3	14	14	T	63	67
03H041	ins	chr13:28608254	chr13:28608254	FLT3	FLT3	14	14	T	63	75
05H034	fusion	chr5:176563031	chr11:3735224	NSD1	NUP98	3	19	F	na	133
05H034	fusion	chr5:176662822	chr11:3765738	NUP98	NSD1	12	7	T	na	45
05H034	fusion	chr5:176662822	chr11:3774545	NUP98	NSD1	11	7	T	na	10
05H163	fusion	chr5:176639196	chr11:3756553	NSD1	NUP98	6	13	T	na	40
05H163	fusion	chr5:176662822	chr11:3765738	NUP98	NSD1	12	7	T	na	19
05H163	fusion	chr5:176662822	chr11:3774545	NUP98	NSD1	11	7	T	na	5
08H049	fusion	chr5:176639196	chr11:3756553	NSD1	NUP98	6	13	T	na	44
08H049	fusion	chr5:176662822	chr11:3765738	NUP98	NSD1	12	7	T	na	35
08H049	fusion	chr5:176662822	chr11:3774545	NUP98	NSD1	11	7	T	na	16
08H049	ITD	chr13:28608128	chr13:28608267	FLT3	FLT3	15	14	T	57	85
10H038	fusion	chr5:176662822	chr11:3765738	NUP98	NSD1	12	7	T	na	49
10H038	fusion	chr5:176662822	chr11:3774545	NUP98	NSD1	11	7	T	na	18
11H027	fusion	chr5:176662822	chr11:3765738	NUP98	NSD1	12	7	T	na	26
11H027	ins	chr5:170837543	chr5:170837543	NPM1	NPM1	11	11	F	4	6
11H027	ITD	chr13:28608232	chr13:28608251	FLT3	FLT3	14	14	T	21	111
11H160	fusion	chr5:176662822	chr11:3765738	NUP98	NSD1	12	7	T	na	35
11H160	fusion	chr5:176662822	chr11:3774545	NUP98	NSD1	11	7	T	na	7
11H160	ins	chr13:28608252	chr13:28608252	FLT3	FLT3	14	14	T	36	116
11H160	ITD	chr13:28608219	chr13:28608308	FLT3	FLT3	14	14	T	93	11
11H160	ITD	chr13:28608238	chr13:28608308	FLT3	FLT3	14	14	T	72	27
11H160	ITD	chr13:28608239	chr13:28608312	FLT3	FLT3	14	14	T	75	31
02H060	ins	chr19:33793032	chr19:33793032	CEBPA	CEBPA	1	1	F	4	36
04H048	del	chr19:33793101	chr19:33793102	CEBPA	CEBPA	1	1	F	1	106
04H048	ins	chr19:33792378	chr19:33792378	CEBPA	CEBPA	1	1	T	3	99
04H048	ins	chr19:33792731	chr19:33792731	CEBPA	CEBPA	1	1	T	6	37
05H078	del	chr19:33793090	chr19:33793091	CEBPA	CEBPA	1	1	F	1	183

05H078	ins	chr19:33792375	chr19:33792375	CEBPA	CEBPA	1	1	T	3	152
06H026	del	chr19:33793115	chr19:33793117	CEBPA	CEBPA	1	1	F	2	144
06H026	ITD	chr13:28608128	chr13:28608272	FLT3	FLT3	15	14	T	57	17
06H026	ITD	chr19:33792361	chr19:33792383	CEBPA	CEBPA	1	1	T	24	132
07H020	del	chr19:33791526	chr19:33791528	CEBPA	CEBPA	1	1	na	2	6
07H020	del	chr19:33792387	chr19:33792405	CEBPA	CEBPA	1	1	T	18	161
07H020	ins	chr19:33793252	chr19:33793252	CEBPA	CEBPA	1	1	F	1	49
08H048	del	chr19:33793022	chr19:33793044	CEBPA	CEBPA	1	1	F	22	5
08H048	ins	chr19:33792390	chr19:33792390	CEBPA	CEBPA	1	1	T	3	248
08H048	ins	chr19:33792393	chr19:33792393	CEBPA	CEBPA	1	1	T	3	7
08H048	ins	chr19:33793231	chr19:33793231	CEBPA	CEBPA	1	1	F	5	184
08H048	ITD	chr13:28608260	chr13:28608279	FLT3	FLT3	14	14	T	21	48
08H065	repeat_expansion	chr19:33792384	chr19:33792384	CEBPA	CEBPA	1	1	T	3	128
08H082	ins	chr13:28608230	chr13:28608230	FLT3	FLT3	14	14	T	18	249
08H082	ins	chr19:33793206	chr19:33793206	CEBPA	CEBPA	1	1	F	2	34
08H082	ins	chr5:170837546	chr5:170837546	NPM1	NPM1	11	11	F	4	193
08H082	ITD	chr13:28608246	chr13:28608321	FLT3	FLT3	14	14	T	75	10
10H089	del	chr19:33793111	chr19:33793112	CEBPA	CEBPA	1	1	F	1	348
10H089	ins	chr19:33792381	chr19:33792381	CEBPA	CEBPA	1	1	T	3	126
12H039	del	chr19:33792968	chr19:33792982	CEBPA	CEBPA	1	1	F	14	91
12H175	ins	chr19:33792381	chr19:33792381	CEBPA	CEBPA	1	1	T	3	147
13H058	del	chr19:33792763	chr19:33792765	CEBPA	CEBPA	1	1	F	2	4
13H058	del	chr19:33793047	chr19:33793048	CEBPA	CEBPA	1	1	F	1	492
13H058	ins	chr19:33792381	chr19:33792381	CEBPA	CEBPA	1	1	T	3	138
09H106	ins	chr13:28608227	chr13:28608227	FLT3	FLT3	14	14	T	27	109
09H106	PTD	chr11:118339490	chr11:118353209	KMT2A	KMT2A	2	8	T	3654	43

cohorts:
CBF-AML
MLL-F
NUP98-ND1
CEBPA
MLL-PTD

Table S5. Aberrant splicing variants previously identified in AML patients detected by TAP in Leucegene samples

sample	event	chrom1	coord1	coord2	event	size	exon1	exon2	in_frame	support_reads
03H065	CD13-Vc	chr15	90347848	90348353	novel_donor	44	5	4	F	13
03H065	FLT3-Va	chr13	28623598	28624232	novel_acceptor	76	8	6	F	22
03H065	FLT3-Va	chr13	28623598	28624232	skipped_exon	140	8	6	F	22
03H065	FLT3-Vb	chr13	28624359	28631497	novel_donor	13	6	4	F	7
03H065	FLT3-Vb	chr13	28624359	28631497	skipped_exon	130	6	4	F	7
03H065	NOTCH2-Va	chr1	120497855	120506197	skipped_exon	111	13	11	T	6
03H065	NOTCH2-Vb	chr1	120484377	120493347	skipped_exon	273	18	15	T	11
03H083	FLT3-Va	chr13	28623598	28624232	novel_acceptor	76	8	6	F	25
03H083	FLT3-Va	chr13	28623598	28624232	skipped_exon	140	8	6	F	25
03H083	NOTCH2-Vb	chr1	120484377	120493347	skipped_exon	273	18	15	T	5
03H095	CD13-Vc	chr15	90347848	90348353	novel_donor	44	5	4	F	12
03H095	FLT3-Va	chr13	28623598	28624232	novel_acceptor	76	8	6	F	15
03H095	FLT3-Va	chr13	28623598	28624232	skipped_exon	140	8	6	F	15
03H095	FLT3-Vc	chr13	28623598	28631497	skipped_exon	398	8	4	F	4
03H095	NOTCH2-Vb	chr1	120484377	120493347	skipped_exon	273	18	15	T	13
03H109	CD13-Vc	chr15	90347848	90348353	novel_donor	44	5	4	F	5
03H109	FLT3-Va	chr13	28623598	28624232	novel_acceptor	76	8	6	F	21
03H109	FLT3-Va	chr13	28623598	28624232	skipped_exon	140	8	6	F	21
03H112	CD13-Vc	chr15	90347848	90348353	novel_donor	44	5	4	F	12
03H112	FLT3-Va	chr13	28623598	28624232	novel_acceptor	76	8	6	F	6
03H112	FLT3-Va	chr13	28623598	28624232	skipped_exon	140	8	6	F	6
04H030	FLT3-Va	chr13	28623598	28624232	novel_acceptor	76	8	6	F	21
04H030	FLT3-Va	chr13	28623598	28624232	skipped_exon	140	8	6	F	21
04H030	FLT3-Vb	chr13	28624359	28631497	novel_donor	13	6	4	F	5
04H030	FLT3-Vb	chr13	28624359	28631497	skipped_exon	130	6	4	F	5
04H030	NOTCH2-Va	chr1	120497855	120506197	skipped_exon	111	13	11	T	5
04H030	NOTCH2-Vb	chr1	120484377	120493347	skipped_exon	273	18	15	T	10
04H061	CD13-Va	chr15	90347848	90348552	skipped_exon	140	5	3	F	8
04H061	CD13-Vc	chr15	90347848	90348353	novel_donor	44	5	4	F	11
04H061	FLT3-Va	chr13	28623598	28624232	novel_acceptor	76	8	6	F	12
04H061	FLT3-Va	chr13	28623598	28624232	skipped_exon	140	8	6	F	12

04H061	NOTCH2-Va	chr1	120497855	120506197	skipped_exon	111	13	11	T	25
04H061	NOTCH2-Vb	chr1	120484377	120493347	skipped_exon	273	18	15	T	48
04H091	FLT3-Va	chr13	28623598	28624232	novel_acceptor	76	8	6	F	12
04H091	FLT3-Va	chr13	28623598	28624232	skipped_exon	140	8	6	F	12
05H042	FLT3-Va	chr13	28623598	28624232	novel_acceptor	76	8	6	F	37
05H042	FLT3-Va	chr13	28623598	28624232	skipped_exon	140	8	6	F	37
05H099	CD13-Va	chr15	90347848	90348552	skipped_exon	140	5	3	F	22
05H099	CD13-Vc	chr15	90347848	90348353	novel_donor	44	5	4	F	16
05H099	FLT3-Va	chr13	28623598	28624232	novel_acceptor	76	8	6	F	32
05H099	FLT3-Va	chr13	28623598	28624232	skipped_exon	140	8	6	F	32
05H099	FLT3-Vb	chr13	28624359	28631497	novel_donor	13	6	4	F	6
05H099	FLT3-Vb	chr13	28624359	28631497	skipped_exon	130	6	4	F	6
05H099	NOTCH2-Va	chr1	120497855	120506197	skipped_exon	111	13	11	T	16
05H099	NOTCH2-Vb	chr1	120484377	120493347	skipped_exon	273	18	15	T	19
05H113	CD13-Vc	chr15	90347848	90348353	novel_donor	44	5	4	F	15
05H113	FLT3-Va	chr13	28623598	28624232	novel_acceptor	76	8	6	F	9
05H113	FLT3-Va	chr13	28623598	28624232	skipped_exon	140	8	6	F	9
05H113	NOTCH2-Va	chr1	120497855	120506197	skipped_exon	111	13	11	T	8
05H113	NOTCH2-Vb	chr1	120484377	120493347	skipped_exon	273	18	15	T	13
05H118	FLT3-Va	chr13	28623598	28624232	novel_acceptor	76	8	6	F	30
05H118	FLT3-Va	chr13	28623598	28624232	skipped_exon	140	8	6	F	30
05H136	CD13-Va	chr15	90347848	90348552	skipped_exon	140	5	3	F	47
05H136	CD13-Vc	chr15	90347848	90348353	novel_donor	44	5	4	F	31
05H136	FLT3-Va	chr13	28623598	28624232	novel_acceptor	76	8	6	F	59
05H136	FLT3-Va	chr13	28623598	28624232	skipped_exon	140	8	6	F	59
05H136	FLT3-Vb	chr13	28624359	28631497	novel_donor	13	6	4	F	8
05H136	FLT3-Vb	chr13	28624359	28631497	skipped_exon	130	6	4	F	8
05H136	NOTCH2-Va	chr1	120497855	120506197	skipped_exon	111	13	11	T	27
05H136	NOTCH2-Vb	chr1	120484377	120493347	skipped_exon	273	18	15	T	50
05H184	FLT3-Va	chr13	28623598	28624232	novel_acceptor	76	8	6	F	43
05H184	FLT3-Va	chr13	28623598	28624232	skipped_exon	140	8	6	F	43
05H184	FLT3-Vb	chr13	28624359	28631497	novel_donor	13	6	4	F	18
05H184	FLT3-Vb	chr13	28624359	28631497	skipped_exon	130	6	4	F	18
05H184	NOTCH2-Vb	chr1	120484377	120493347	skipped_exon	273	18	15	T	11

06H020	CD13-Va	chr15	90347848	90348552	skipped_exon	140	5	3	F	6
06H020	CD13-Vc	chr15	90347848	90348353	novel_donor	44	5	4	F	9
06H020	FLT3-Va	chr13	28623598	28624232	novel_acceptor	76	8	6	F	21
06H020	FLT3-Va	chr13	28623598	28624232	skipped_exon	140	8	6	F	21
06H020	NOTCH2-Vb	chr1	120484377	120493347	skipped_exon	273	18	15	T	7
06H035	CD13-Va	chr15	90347848	90348552	skipped_exon	140	5	3	F	10
06H035	CD13-Vc	chr15	90347848	90348353	novel_donor	44	5	4	F	15
06H035	FLT3-Va	chr13	28623598	28624232	novel_acceptor	76	8	6	F	14
06H035	FLT3-Va	chr13	28623598	28624232	skipped_exon	140	8	6	F	14
06H035	NOTCH2-Va	chr1	120497855	120506197	skipped_exon	111	13	11	T	17
06H035	NOTCH2-Vb	chr1	120484377	120493347	skipped_exon	273	18	15	T	33
06H115	CD13-Va	chr15	90347848	90348552	skipped_exon	140	5	3	F	11
06H115	CD13-Vc	chr15	90347848	90348353	novel_donor	44	5	4	F	20
06H115	FLT3-Va	chr13	28623598	28624232	novel_acceptor	76	8	6	F	12
06H115	FLT3-Va	chr13	28623598	28624232	skipped_exon	140	8	6	F	12
06H115	NOTCH2-Vb	chr1	120484377	120493347	skipped_exon	273	18	15	T	16
07H099	CD13-Va	chr15	90347848	90348552	skipped_exon	140	5	3	F	13
07H099	CD13-Vc	chr15	90347848	90348353	novel_donor	44	5	4	F	6
07H099	NOTCH2-Va	chr1	120497855	120506197	skipped_exon	111	13	11	T	10
07H099	NOTCH2-Vb	chr1	120484377	120493347	skipped_exon	273	18	15	T	25
07H137	FLT3-Va	chr13	28623598	28624232	novel_acceptor	76	8	6	F	45
07H137	FLT3-Va	chr13	28623598	28624232	skipped_exon	140	8	6	F	45
07H137	FLT3-Vb	chr13	28624359	28631497	novel_donor	13	6	4	F	7
07H137	FLT3-Vb	chr13	28624359	28631497	skipped_exon	130	6	4	F	7
07H137	NOTCH2-Vb	chr1	120484377	120493347	skipped_exon	273	18	15	T	10
07H144	CD13-Va	chr15	90347848	90348552	skipped_exon	140	5	3	F	8
07H144	CD13-Vc	chr15	90347848	90348353	novel_donor	44	5	4	F	12
07H144	FLT3-Va	chr13	28623598	28624232	novel_acceptor	76	8	6	F	47
07H144	FLT3-Va	chr13	28623598	28624232	skipped_exon	140	8	6	F	47
07H144	FLT3-Vb	chr13	28624359	28631497	novel_donor	13	6	4	F	4
07H144	FLT3-Vb	chr13	28624359	28631497	skipped_exon	130	6	4	F	4
07H144	NOTCH2-Va	chr1	120497855	120506197	skipped_exon	111	13	11	T	11
07H144	NOTCH2-Vb	chr1	120484377	120493347	skipped_exon	273	18	15	T	23
08H034	CD13-Va	chr15	90347848	90348552	skipped_exon	140	5	3	F	8

08H034	CD13-Vc	chr15	90347848	90348353	novel_donor	44	5	4	F	41
08H034	FLT3-Va	chr13	28623598	28624232	novel_acceptor	76	8	6	F	32
08H034	FLT3-Va	chr13	28623598	28624232	skipped_exon	140	8	6	F	32
08H034	FLT3-Vb	chr13	28624359	28631497	novel_donor	13	6	4	F	8
08H034	FLT3-Vb	chr13	28624359	28631497	skipped_exon	130	6	4	F	8
08H034	NOTCH2-Va	chr1	120497855	120506197	skipped_exon	111	13	11	T	23
08H034	NOTCH2-Vb	chr1	120484377	120493347	skipped_exon	273	18	15	T	23
08H042	FLT3-Va	chr13	28623598	28624232	novel_acceptor	76	8	6	F	22
08H042	FLT3-Va	chr13	28623598	28624232	skipped_exon	140	8	6	F	22
08H042	NOTCH2-Va	chr1	120497855	120506197	skipped_exon	111	13	11	T	4
08H042	NOTCH2-Vb	chr1	120484377	120493347	skipped_exon	273	18	15	T	5
08H072	CD13-Vc	chr15	90347848	90348353	novel_donor	44	5	4	F	11
08H072	FLT3-Va	chr13	28623598	28624232	novel_acceptor	76	8	6	F	37
08H072	FLT3-Va	chr13	28623598	28624232	skipped_exon	140	8	6	F	37
08H072	FLT3-Vb	chr13	28624359	28631497	novel_donor	13	6	4	F	5
08H072	FLT3-Vb	chr13	28624359	28631497	skipped_exon	130	6	4	F	5
08H072	NOTCH2-Va	chr1	120497855	120506197	skipped_exon	111	13	11	T	11
08H072	NOTCH2-Vb	chr1	120484377	120493347	skipped_exon	273	18	15	T	5
08H081	CD13-Va	chr15	90347848	90348552	skipped_exon	140	5	3	F	27
08H081	CD13-Vc	chr15	90347848	90348353	novel_donor	44	5	4	F	14
08H081	FLT3-Va	chr13	28623598	28624232	novel_acceptor	76	8	6	F	28
08H081	FLT3-Va	chr13	28623598	28624232	skipped_exon	140	8	6	F	28
08H081	FLT3-Vb	chr13	28624359	28631497	novel_donor	13	6	4	F	5
08H081	FLT3-Vb	chr13	28624359	28631497	skipped_exon	130	6	4	F	5
08H081	NOTCH2-Va	chr1	120497855	120506197	skipped_exon	111	13	11	T	16
08H081	NOTCH2-Vb	chr1	120484377	120493347	skipped_exon	273	18	15	T	38
08H099	CD13-Va	chr15	90347848	90348552	skipped_exon	140	5	3	F	23
08H099	CD13-Vc	chr15	90347848	90348353	novel_donor	44	5	4	F	22
08H099	FLT3-Va	chr13	28623598	28624232	novel_acceptor	76	8	6	F	13
08H099	FLT3-Va	chr13	28623598	28624232	skipped_exon	140	8	6	F	13
08H099	NOTCH2-Va	chr1	120497855	120506197	skipped_exon	111	13	11	T	27
08H099	NOTCH2-Vb	chr1	120484377	120493347	skipped_exon	273	18	15	T	25
09H016	CD13-Vc	chr15	90347848	90348353	novel_donor	44	5	4	F	9
09H016	FLT3-Va	chr13	28623598	28624232	novel_acceptor	76	8	6	F	30

09H016	FLT3-Va	chr13	28623598	28624232	skipped_exon	140	8	6	F	30
09H040	FLT3-Va	chr13	28623598	28624232	novel_acceptor	76	8	6	F	24
09H040	FLT3-Va	chr13	28623598	28624232	skipped_exon	140	8	6	F	24
09H066	CD13-Va	chr15	90347848	90348552	skipped_exon	140	5	3	F	33
09H066	CD13-Vc	chr15	90347848	90348353	novel_donor	44	5	4	F	30
09H066	FLT3-Va	chr13	28623598	28624232	novel_acceptor	76	8	6	F	15
09H066	FLT3-Va	chr13	28623598	28624232	skipped_exon	140	8	6	F	15
09H066	NOTCH2-Va	chr1	120497855	120506197	skipped_exon	111	13	11	T	33
09H066	NOTCH2-Vb	chr1	120484377	120493347	skipped_exon	273	18	15	T	33
10H008	CD13-Va	chr15	90347848	90348552	skipped_exon	140	5	3	F	10
10H008	CD13-Vc	chr15	90347848	90348353	novel_donor	44	5	4	F	19
10H008	FLT3-Va	chr13	28623598	28624232	novel_acceptor	76	8	6	F	18
10H008	FLT3-Va	chr13	28623598	28624232	skipped_exon	140	8	6	F	18
10H008	NOTCH2-Vb	chr1	120484377	120493347	skipped_exon	273	18	15	T	9
10H030	CD13-Vc	chr15	90347848	90348353	novel_donor	44	5	4	F	9
10H030	FLT3-Va	chr13	28623598	28624232	novel_acceptor	76	8	6	F	70
10H030	FLT3-Va	chr13	28623598	28624232	skipped_exon	140	8	6	F	70
10H030	FLT3-Vb	chr13	28624359	28631497	novel_donor	13	6	4	F	4
10H030	FLT3-Vb	chr13	28624359	28631497	skipped_exon	130	6	4	F	4
10H030	NOTCH2-Va	chr1	120497855	120506197	skipped_exon	111	13	11	T	23
10H030	NOTCH2-Vb	chr1	120484377	120493347	skipped_exon	273	18	15	T	26
10H119	CD13-Vc	chr15	90347848	90348353	novel_donor	44	5	4	F	4
10H119	FLT3-Va	chr13	28623598	28624232	novel_acceptor	76	8	6	F	42
10H119	FLT3-Va	chr13	28623598	28624232	skipped_exon	140	8	6	F	42
10H119	FLT3-Vb	chr13	28624359	28631497	novel_donor	13	6	4	F	8
10H119	FLT3-Vb	chr13	28624359	28631497	skipped_exon	130	6	4	F	8
10H119	NOTCH2-Va	chr1	120497855	120506197	skipped_exon	111	13	11	T	4
10H119	NOTCH2-Vb	chr1	120484377	120493347	skipped_exon	273	18	15	T	8
11H022	CD13-Va	chr15	90347848	90348552	skipped_exon	140	5	3	F	18
11H022	CD13-Vc	chr15	90347848	90348353	novel_donor	44	5	4	F	27
11H022	FLT3-Va	chr13	28623598	28624232	novel_acceptor	76	8	6	F	13
11H022	FLT3-Va	chr13	28623598	28624232	skipped_exon	140	8	6	F	13
11H022	NOTCH2-Va	chr1	120497855	120506197	skipped_exon	111	13	11	T	11
11H022	NOTCH2-Vb	chr1	120484377	120493347	skipped_exon	273	18	15	T	18

11H104	CD13-Va	chr15	90347848	90348552	skipped_exon	140	5	3	F	21
11H104	CD13-Vc	chr15	90347848	90348353	novel_donor	44	5	4	F	47
11H104	FLT3-Va	chr13	28623598	28624232	novel_acceptor	76	8	6	F	48
11H104	FLT3-Va	chr13	28623598	28624232	skipped_exon	140	8	6	F	48
11H104	FLT3-Vb	chr13	28624359	28631497	novel_donor	13	6	4	F	9
11H104	FLT3-Vb	chr13	28624359	28631497	skipped_exon	130	6	4	F	9
11H104	NOTCH2-Va	chr1	120497855	120506197	skipped_exon	111	13	11	T	53
11H104	NOTCH2-Vb	chr1	120484377	120493347	skipped_exon	273	18	15	T	55
11H107	FLT3-Va	chr13	28623598	28624232	novel_acceptor	76	8	6	F	42
11H107	FLT3-Va	chr13	28623598	28624232	skipped_exon	140	8	6	F	42
11H107	FLT3-Vb	chr13	28624359	28631497	novel_donor	13	6	4	F	9
11H107	FLT3-Vb	chr13	28624359	28631497	skipped_exon	130	6	4	F	9
11H179	CD13-Va	chr15	90347848	90348552	skipped_exon	140	5	3	F	31
11H179	CD13-Vc	chr15	90347848	90348353	novel_donor	44	5	4	F	65
11H179	CD13-Vc	chr15	90347848	90348357	novel_donor	48	5	4	T	6
11H179	FLT3-Va	chr13	28623598	28624232	novel_acceptor	76	8	6	F	32
11H179	FLT3-Va	chr13	28623598	28624232	skipped_exon	140	8	6	F	32
11H179	FLT3-Vb	chr13	28624359	28631497	novel_donor	13	6	4	F	5
11H179	FLT3-Vb	chr13	28624359	28631497	skipped_exon	130	6	4	F	5
11H179	NOTCH2-Va	chr1	120497855	120506197	skipped_exon	111	13	11	T	35
11H179	NOTCH2-Vb	chr1	120484377	120493347	skipped_exon	273	18	15	T	37
12H042	CD13-Va	chr15	90347848	90348552	skipped_exon	140	5	3	F	10
12H042	CD13-Vc	chr15	90347848	90348353	novel_donor	44	5	4	F	24
12H042	FLT3-Va	chr13	28623598	28624232	novel_acceptor	76	8	6	F	30
12H042	FLT3-Va	chr13	28623598	28624232	skipped_exon	140	8	6	F	30
12H042	FLT3-Vb	chr13	28624359	28631497	novel_donor	13	6	4	F	5
12H042	FLT3-Vb	chr13	28624359	28631497	skipped_exon	130	6	4	F	5
12H042	NOTCH2-Va	chr1	120497855	120506197	skipped_exon	111	13	11	T	14
12H042	NOTCH2-Vb	chr1	120484377	120493347	skipped_exon	273	18	15	T	29
12H044	CD13-Va	chr15	90347848	90348552	skipped_exon	140	5	3	F	18
12H044	CD13-Vc	chr15	90347848	90348353	novel_donor	44	5	4	F	23
12H044	FLT3-Va	chr13	28623598	28624232	novel_acceptor	76	8	6	F	48
12H044	FLT3-Va	chr13	28623598	28624232	skipped_exon	140	8	6	F	48
12H044	FLT3-Vb	chr13	28624359	28631497	novel_donor	13	6	4	F	5

12H044	FLT3-Vb	chr13	28624359	28631497	skipped_exon	130	6	4	F	5
12H044	NOTCH2-Va	chr1	120497855	120506197	skipped_exon	111	13	11	T	7
12H044	NOTCH2-Vb	chr1	120484377	120493347	skipped_exon	273	18	15	T	37
12H045	CD13-Vc	chr15	90347848	90348353	novel_donor	44	5	4	F	7
12H045	FLT3-Va	chr13	28623598	28624232	novel_acceptor	76	8	6	F	18
12H045	FLT3-Va	chr13	28623598	28624232	skipped_exon	140	8	6	F	18
12H045	FLT3-Vb	chr13	28624359	28631497	novel_donor	13	6	4	F	5
12H045	FLT3-Vb	chr13	28624359	28631497	skipped_exon	130	6	4	F	5
12H045	NOTCH2-Va	chr1	120497855	120506197	skipped_exon	111	13	11	T	4
12H045	NOTCH2-Vb	chr1	120484377	120493347	skipped_exon	273	18	15	T	17
12H098	CD13-Vc	chr15	90347848	90348353	novel_donor	44	5	4	F	15
12H098	FLT3-Va	chr13	28623598	28624232	novel_acceptor	76	8	6	F	7
12H098	FLT3-Va	chr13	28623598	28624232	skipped_exon	140	8	6	F	7
12H098	FLT3-Vb	chr13	28624359	28631497	novel_donor	13	6	4	F	5
12H098	FLT3-Vb	chr13	28624359	28631497	skipped_exon	130	6	4	F	5
12H098	NOTCH2-Va	chr1	120497855	120506197	skipped_exon	111	13	11	T	21
12H098	NOTCH2-Vb	chr1	120484377	120493347	skipped_exon	273	18	15	T	22
12H165	CD13-Va	chr15	90347848	90348552	skipped_exon	140	5	3	F	9
12H165	CD13-Vc	chr15	90347848	90348353	novel_donor	44	5	4	F	21
12H165	FLT3-Va	chr13	28623598	28624232	novel_acceptor	76	8	6	F	52
12H165	FLT3-Va	chr13	28623598	28624232	skipped_exon	140	8	6	F	52
12H165	FLT3-Vb	chr13	28624359	28631497	novel_donor	13	6	4	F	6
12H165	FLT3-Vb	chr13	28624359	28631497	skipped_exon	130	6	4	F	6
12H165	NOTCH2-Vb	chr1	120484377	120493347	skipped_exon	273	18	15	T	31
12H166	FLT3-Va	chr13	28623598	28624232	novel_acceptor	76	8	6	F	24
12H166	FLT3-Va	chr13	28623598	28624232	skipped_exon	140	8	6	F	24
12H166	NOTCH2-Va	chr1	120497855	120506197	skipped_exon	111	13	11	T	9
12H166	NOTCH2-Vb	chr1	120484377	120493347	skipped_exon	273	18	15	T	16
12H180	CD13-Vc	chr15	90347848	90348353	novel_donor	44	5	4	F	14
12H180	FLT3-Va	chr13	28623598	28624232	novel_acceptor	76	8	6	F	34
12H180	FLT3-Va	chr13	28623598	28624232	skipped_exon	140	8	6	F	34
12H180	FLT3-Vb	chr13	28624359	28631497	novel_donor	13	6	4	F	7
12H180	FLT3-Vb	chr13	28624359	28631497	skipped_exon	130	6	4	F	7
12H180	NOTCH2-Va	chr1	120497855	120506197	skipped_exon	111	13	11	T	9

12H180	NOTCH2-Vb	chr1	120484377	120493347	skipped_exon	273	18	15	T	12
12H183	CD13-Vc	chr15	90347848	90348353	novel_donor	44	5	4	F	4
12H183	FLT3-Va	chr13	28623598	28624232	novel_acceptor	76	8	6	F	40
12H183	FLT3-Va	chr13	28623598	28624232	skipped_exon	140	8	6	F	40
12H183	FLT3-Vb	chr13	28624359	28631497	novel_donor	13	6	4	F	6
12H183	FLT3-Vb	chr13	28624359	28631497	skipped_exon	130	6	4	F	6
12H183	NOTCH2-Va	chr1	120497855	120506197	skipped_exon	111	13	11	T	10
12H183	NOTCH2-Vb	chr1	120484377	120493347	skipped_exon	273	18	15	T	11
13H066	CD13-Vc	chr15	90347848	90348353	novel_donor	44	5	4	F	14
13H066	FLT3-Va	chr13	28623598	28624232	novel_acceptor	76	8	6	F	10
13H066	FLT3-Va	chr13	28623598	28624232	skipped_exon	140	8	6	F	10
13H066	NOTCH2-Vb	chr1	120484377	120493347	skipped_exon	273	18	15	T	10
13H120	CD13-Va	chr15	90347848	90348552	skipped_exon	140	5	3	F	13
13H120	CD13-Vc	chr15	90347848	90348353	novel_donor	44	5	4	F	19
13H120	FLT3-Va	chr13	28623598	28624232	novel_acceptor	76	8	6	F	17
13H120	FLT3-Va	chr13	28623598	28624232	skipped_exon	140	8	6	F	17
13H120	NOTCH2-Va	chr1	120497855	120506197	skipped_exon	111	13	11	T	12
13H120	NOTCH2-Vb	chr1	120484377	120493347	skipped_exon	273	18	15	T	39
13H169	CD13-Vc	chr15	90347848	90348353	novel_donor	44	5	4	F	10
13H169	FLT3-Va	chr13	28623598	28624232	novel_acceptor	76	8	6	F	35
13H169	FLT3-Va	chr13	28623598	28624232	skipped_exon	140	8	6	F	35
13H169	FLT3-Vb	chr13	28624359	28631497	novel_donor	13	6	4	F	4
13H169	FLT3-Vb	chr13	28624359	28631497	skipped_exon	130	6	4	F	4
13H169	NOTCH2-Va	chr1	120497855	120506197	skipped_exon	111	13	11	T	11
13H169	NOTCH2-Vb	chr1	120484377	120493347	skipped_exon	273	18	15	T	16
01H001	CD13-Va	chr15	90347848	90348552	skipped_exon	140	5	3	F	5
01H001	FLT3-Va	chr13	28623598	28624232	novel_acceptor	76	8	6	F	13
01H001	FLT3-Va	chr13	28623598	28624232	skipped_exon	140	8	6	F	13
02H017	FLT3-Va	chr13	28623598	28624232	novel_acceptor	76	8	6	F	10
02H017	FLT3-Va	chr13	28623598	28624232	skipped_exon	140	8	6	F	10
02H032	FLT3-Va	chr13	28623598	28624232	novel_acceptor	76	8	6	F	29
02H032	FLT3-Va	chr13	28623598	28624232	skipped_exon	140	8	6	F	29
03H067	FLT3-Va	chr13	28623598	28624232	novel_acceptor	76	8	6	F	25
03H067	FLT3-Va	chr13	28623598	28624232	skipped_exon	140	8	6	F	25

03H067	FLT3-Vb	chr13	28624359	28631497	novel_donor	13	6	4	F	5
03H067	FLT3-Vb	chr13	28624359	28631497	skipped_exon	130	6	4	F	5
03H067	NOTCH2-Vb	chr1	120484377	120493347	skipped_exon	273	18	15	T	14
04H041	FLT3-Va	chr13	28623598	28624232	novel_acceptor	76	8	6	F	22
04H041	FLT3-Va	chr13	28623598	28624232	skipped_exon	140	8	6	F	22
04H041	NOTCH2-Va	chr1	120497855	120506197	skipped_exon	111	13	11	T	10
04H041	NOTCH2-Vb	chr1	120484377	120493347	skipped_exon	273	18	15	T	46
04H080	FLT3-Va	chr13	28623598	28624232	novel_acceptor	76	8	6	F	13
04H080	FLT3-Va	chr13	28623598	28624232	skipped_exon	140	8	6	F	13
04H080	NOTCH2-Vb	chr1	120484377	120493347	skipped_exon	273	18	15	T	7
04H121	FLT3-Va	chr13	28623598	28624232	novel_acceptor	76	8	6	F	71
04H121	FLT3-Va	chr13	28623598	28624232	skipped_exon	140	8	6	F	71
04H121	FLT3-Vc	chr13	28623598	28631497	skipped_exon	398	8	4	F	5
05H025	FLT3-Va	chr13	28623598	28624232	novel_acceptor	76	8	6	F	28
05H025	FLT3-Va	chr13	28623598	28624232	skipped_exon	140	8	6	F	28
05H025	FLT3-Vb	chr13	28624359	28631497	novel_donor	13	6	4	F	9
05H025	FLT3-Vb	chr13	28624359	28631497	skipped_exon	130	6	4	F	9
05H025	NOTCH2-Va	chr1	120497855	120506197	skipped_exon	111	13	11	T	22
05H025	NOTCH2-Vb	chr1	120484377	120493347	skipped_exon	273	18	15	T	42
05H066	CD13-Va	chr15	90347848	90348552	skipped_exon	140	5	3	F	6
05H066	CD13-Vc	chr15	90347848	90348353	novel_donor	44	5	4	F	15
05H066	FLT3-Va	chr13	28623598	28624232	novel_acceptor	76	8	6	F	34
05H066	FLT3-Va	chr13	28623598	28624232	skipped_exon	140	8	6	F	34
05H066	FLT3-Vb	chr13	28624359	28631497	novel_donor	13	6	4	F	6
05H066	FLT3-Vb	chr13	28624359	28631497	skipped_exon	130	6	4	F	6
05H066	NOTCH2-Va	chr1	120497855	120506197	skipped_exon	111	13	11	T	10
05H066	NOTCH2-Vb	chr1	120484377	120493347	skipped_exon	273	18	15	T	26
05H128	FLT3-Va	chr13	28623598	28624232	novel_acceptor	76	8	6	F	29
05H128	FLT3-Va	chr13	28623598	28624232	skipped_exon	140	8	6	F	29
05H128	FLT3-Vb	chr13	28624359	28631497	novel_donor	13	6	4	F	7
05H128	FLT3-Vb	chr13	28624359	28631497	skipped_exon	130	6	4	F	7
05H128	NOTCH2-Vb	chr1	120484377	120493347	skipped_exon	273	18	15	T	58
06H066	CD13-Vc	chr15	90347848	90348353	novel_donor	44	5	4	F	5
06H066	FLT3-Va	chr13	28623598	28624232	novel_acceptor	76	8	6	F	24

06H066	FLT3-Va	chr13	28623598	28624232	skipped_exon	140	8	6	F	24
06H066	FLT3-Vb	chr13	28624359	28631497	novel_donor	13	6	4	F	14
06H066	FLT3-Vb	chr13	28624359	28631497	skipped_exon	130	6	4	F	14
06H066	FLT3-Vc	chr13	28623598	28631497	skipped_exon	398	8	4	F	4
06H066	NOTCH2-Va	chr1	120497855	120506197	skipped_exon	111	13	11	T	7
06H066	NOTCH2-Vb	chr1	120484377	120493347	skipped_exon	273	18	15	T	12
06H088	FLT3-Va	chr13	28623598	28624232	novel_acceptor	76	8	6	F	18
06H088	FLT3-Va	chr13	28623598	28624232	skipped_exon	140	8	6	F	18
06H088	NOTCH2-Vb	chr1	120484377	120493347	skipped_exon	273	18	15	T	11
06H117	CD13-Vc	chr15	90347848	90348353	novel_donor	44	5	4	F	6
06H117	FLT3-Va	chr13	28623598	28624232	novel_acceptor	76	8	6	F	12
06H117	FLT3-Va	chr13	28623598	28624232	skipped_exon	140	8	6	F	12
06H117	FLT3-Vb	chr13	28624359	28631497	novel_donor	13	6	4	F	5
06H117	FLT3-Vb	chr13	28624359	28631497	skipped_exon	130	6	4	F	5
06H117	NOTCH2-Va	chr1	120497855	120506197	skipped_exon	111	13	11	T	19
06H117	NOTCH2-Vb	chr1	120484377	120493347	skipped_exon	273	18	15	T	52
06H152	CD13-Va	chr15	90347848	90348552	skipped_exon	140	5	3	F	8
06H152	FLT3-Va	chr13	28623598	28624232	novel_acceptor	76	8	6	F	26
06H152	FLT3-Va	chr13	28623598	28624232	skipped_exon	140	8	6	F	26
06H152	FLT3-Vb	chr13	28624359	28631497	novel_donor	13	6	4	F	13
06H152	FLT3-Vb	chr13	28624359	28631497	skipped_exon	130	6	4	F	13
06H152	NOTCH2-Vb	chr1	120484377	120493347	skipped_exon	273	18	15	T	32
07H003	FLT3-Va	chr13	28623598	28624232	novel_acceptor	76	8	6	F	36
07H003	FLT3-Va	chr13	28623598	28624232	skipped_exon	140	8	6	F	36
07H003	FLT3-Vb	chr13	28624359	28631497	novel_donor	13	6	4	F	4
07H003	FLT3-Vb	chr13	28624359	28631497	skipped_exon	130	6	4	F	4
07H003	NOTCH2-Va	chr1	120497855	120506197	skipped_exon	111	13	11	T	6
07H003	NOTCH2-Vb	chr1	120484377	120493347	skipped_exon	273	18	15	T	24
07H041	FLT3-Va	chr13	28623598	28624232	novel_acceptor	76	8	6	F	64
07H041	FLT3-Va	chr13	28623598	28624232	skipped_exon	140	8	6	F	64
07H041	FLT3-Vb	chr13	28624359	28631497	novel_donor	13	6	4	F	14
07H041	FLT3-Vb	chr13	28624359	28631497	skipped_exon	130	6	4	F	14
07H041	NOTCH2-Va	chr1	120497855	120506197	skipped_exon	111	13	11	T	8
07H041	NOTCH2-Vb	chr1	120484377	120493347	skipped_exon	273	18	15	T	25

07H045	FLT3-Va	chr13	28623598	28624232	novel_acceptor	76	8	6	F	60
07H045	FLT3-Va	chr13	28623598	28624232	skipped_exon	140	8	6	F	60
07H045	FLT3-Vb	chr13	28624359	28631497	novel_donor	13	6	4	F	15
07H045	FLT3-Vb	chr13	28624359	28631497	skipped_exon	130	6	4	F	15
07H045	NOTCH2-Va	chr1	120497855	120506197	skipped_exon	111	13	11	T	10
07H045	NOTCH2-Vb	chr1	120484377	120493347	skipped_exon	273	18	15	T	32
08H021	FLT3-Va	chr13	28623598	28624232	novel_acceptor	76	8	6	F	30
08H021	FLT3-Va	chr13	28623598	28624232	skipped_exon	140	8	6	F	30
08H021	NOTCH2-Va	chr1	120497855	120506197	skipped_exon	111	13	11	T	5
08H021	NOTCH2-Vb	chr1	120484377	120493347	skipped_exon	273	18	15	T	14
08H085	FLT3-Va	chr13	28623598	28624232	novel_acceptor	76	8	6	F	35
08H085	FLT3-Va	chr13	28623598	28624232	skipped_exon	140	8	6	F	35
08H085	FLT3-Vb	chr13	28624359	28631497	novel_donor	13	6	4	F	41
08H085	FLT3-Vb	chr13	28624359	28631497	skipped_exon	130	6	4	F	41
08H085	NOTCH2-Va	chr1	120497855	120506197	skipped_exon	111	13	11	T	8
08H085	NOTCH2-Vb	chr1	120484377	120493347	skipped_exon	273	18	15	T	17
08H129	CD13-Vc	chr15	90347848	90348353	novel_donor	44	5	4	F	21
08H129	FLT3-Va	chr13	28623598	28624232	novel_acceptor	76	8	6	F	45
08H129	FLT3-Va	chr13	28623598	28624232	skipped_exon	140	8	6	F	45
08H129	FLT3-Vb	chr13	28624359	28631497	novel_donor	13	6	4	F	8
08H129	FLT3-Vb	chr13	28624359	28631497	skipped_exon	130	6	4	F	8
08H129	NOTCH2-Va	chr1	120497855	120506197	skipped_exon	111	13	11	T	8
08H129	NOTCH2-Vb	chr1	120484377	120493347	skipped_exon	273	18	15	T	36
08H139	FLT3-Va	chr13	28623598	28624232	novel_acceptor	76	8	6	F	74
08H139	FLT3-Va	chr13	28623598	28624232	skipped_exon	140	8	6	F	74
08H139	FLT3-Vb	chr13	28624359	28631497	novel_donor	13	6	4	F	14
08H139	FLT3-Vb	chr13	28624359	28631497	skipped_exon	130	6	4	F	14
08H139	NOTCH2-Va	chr1	120497855	120506197	skipped_exon	111	13	11	T	11
08H139	NOTCH2-Vb	chr1	120484377	120493347	skipped_exon	273	18	15	T	30
09H010	FLT3-Va	chr13	28623598	28624232	novel_acceptor	76	8	6	F	45
09H010	FLT3-Va	chr13	28623598	28624232	skipped_exon	140	8	6	F	45
09H010	FLT3-Vb	chr13	28624359	28631497	novel_donor	13	6	4	F	4
09H010	FLT3-Vb	chr13	28624359	28631497	skipped_exon	130	6	4	F	4
09H010	NOTCH2-Va	chr1	120497855	120506197	skipped_exon	111	13	11	T	10

09H010	NOTCH2-Vb	chr1	120484377	120493347	skipped_exon	273	18	15	T	24
09H018	CD13-Vc	chr15	90347848	90348353	novel_donor	44	5	4	F	14
09H018	NOTCH2-Va	chr1	120497855	120506197	skipped_exon	111	13	11	T	20
09H018	NOTCH2-Vb	chr1	120484377	120493347	skipped_exon	273	18	15	T	29
09H032	FLT3-Va	chr13	28623598	28624232	novel_acceptor	76	8	6	F	63
09H032	FLT3-Va	chr13	28623598	28624232	skipped_exon	140	8	6	F	63
09H032	FLT3-Vb	chr13	28624359	28631497	novel_donor	13	6	4	F	10
09H032	FLT3-Vb	chr13	28624359	28631497	skipped_exon	130	6	4	F	10
09H032	NOTCH2-Vb	chr1	120484377	120493347	skipped_exon	273	18	15	T	27
09H098	FLT3-Va	chr13	28623598	28624232	novel_acceptor	76	8	6	F	19
09H098	FLT3-Va	chr13	28623598	28624232	skipped_exon	140	8	6	F	19
09H098	FLT3-Vb	chr13	28624359	28631497	novel_donor	13	6	4	F	6
09H098	FLT3-Vb	chr13	28624359	28631497	skipped_exon	130	6	4	F	6
09H098	NOTCH2-Vb	chr1	120484377	120493347	skipped_exon	273	18	15	T	18
10H031	CD13-Va	chr15	90347848	90348552	skipped_exon	140	5	3	F	7
10H031	CD13-Vc	chr15	90347848	90348353	novel_donor	44	5	4	F	7
10H031	FLT3-Va	chr13	28623598	28624232	novel_acceptor	76	8	6	F	17
10H031	FLT3-Va	chr13	28623598	28624232	skipped_exon	140	8	6	F	17
10H031	FLT3-Vb	chr13	28624359	28631497	novel_donor	13	6	4	F	4
10H031	FLT3-Vb	chr13	28624359	28631497	skipped_exon	130	6	4	F	4
10H031	NOTCH2-Va	chr1	120497855	120506197	skipped_exon	111	13	11	T	28
10H031	NOTCH2-Vb	chr1	120484377	120493347	skipped_exon	273	18	15	T	45
10H058	FLT3-Va	chr13	28623598	28624232	novel_acceptor	76	8	6	F	33
10H058	FLT3-Va	chr13	28623598	28624232	skipped_exon	140	8	6	F	33
10H058	NOTCH2-Va	chr1	120497855	120506197	skipped_exon	111	13	11	T	19
10H058	NOTCH2-Vb	chr1	120484377	120493347	skipped_exon	273	18	15	T	27
10H127	FLT3-Va	chr13	28623598	28624232	novel_acceptor	76	8	6	F	26
10H127	FLT3-Va	chr13	28623598	28624232	skipped_exon	140	8	6	F	26
10H127	FLT3-Vb	chr13	28624359	28631497	novel_donor	13	6	4	F	5
10H127	FLT3-Vb	chr13	28624359	28631497	skipped_exon	130	6	4	F	5
12H057	CD13-Va	chr15	90347848	90348552	skipped_exon	140	5	3	F	12
12H057	CD13-Vc	chr15	90347848	90348353	novel_donor	44	5	4	F	34
12H057	FLT3-Va	chr13	28623598	28624232	novel_acceptor	76	8	6	F	29
12H057	FLT3-Va	chr13	28623598	28624232	skipped_exon	140	8	6	F	29

12H057	FLT3-Vb	chr13	28624359	28631497	novel_donor	13	6	4	F	7
12H057	FLT3-Vb	chr13	28624359	28631497	skipped_exon	130	6	4	F	7
12H057	NOTCH2-Va	chr1	120497855	120506197	skipped_exon	111	13	11	T	8
12H057	NOTCH2-Vb	chr1	120484377	120493347	skipped_exon	273	18	15	T	23
14H031	FLT3-Va	chr13	28623598	28624232	novel_acceptor	76	8	6	F	49
14H031	FLT3-Va	chr13	28623598	28624232	skipped_exon	140	8	6	F	49
14H031	NOTCH2-Va	chr1	120497855	120506197	skipped_exon	111	13	11	T	21
14H031	NOTCH2-Vb	chr1	120484377	120493347	skipped_exon	273	18	15	T	38
03H041	FLT3-Va	chr13	28623598	28624232	novel_acceptor	76	8	6	F	4
03H041	FLT3-Va	chr13	28623598	28624232	skipped_exon	140	8	6	F	4
05H034	CD13-Vc	chr15	90347848	90348353	novel_donor	44	5	4	F	6
05H034	FLT3-Va	chr13	28623598	28624232	novel_acceptor	76	8	6	F	20
05H034	FLT3-Va	chr13	28623598	28624232	skipped_exon	140	8	6	F	20
05H034	FLT3-Vb	chr13	28624359	28631497	novel_donor	13	6	4	F	5
05H034	FLT3-Vb	chr13	28624359	28631497	skipped_exon	130	6	4	F	5
05H034	NOTCH2-Va	chr1	120497855	120506197	skipped_exon	111	13	11	T	4
05H034	NOTCH2-Vb	chr1	120484377	120493347	skipped_exon	273	18	15	T	15
05H163	CD13-Vc	chr15	90347848	90348353	novel_donor	44	5	4	F	5
05H163	FLT3-Va	chr13	28623598	28624232	novel_acceptor	76	8	6	F	16
05H163	FLT3-Va	chr13	28623598	28624232	skipped_exon	140	8	6	F	16
05H163	NOTCH2-Va	chr1	120497855	120506197	skipped_exon	111	13	11	T	7
05H163	NOTCH2-Vb	chr1	120484377	120493347	skipped_exon	273	18	15	T	16
08H049	FLT3-Va	chr13	28623598	28624232	novel_acceptor	76	8	6	F	23
08H049	FLT3-Va	chr13	28623598	28624232	skipped_exon	140	8	6	F	23
08H049	FLT3-Vb	chr13	28624359	28631497	novel_donor	13	6	4	F	4
08H049	FLT3-Vb	chr13	28624359	28631497	skipped_exon	130	6	4	F	4
08H049	NOTCH2-Va	chr1	120497855	120506197	skipped_exon	111	13	11	T	19
08H049	NOTCH2-Vb	chr1	120484377	120493347	skipped_exon	273	18	15	T	36
10H038	CD13-Vc	chr15	90347848	90348353	novel_donor	44	5	4	F	8
10H038	FLT3-Va	chr13	28623598	28624232	novel_acceptor	76	8	6	F	52
10H038	FLT3-Va	chr13	28623598	28624232	skipped_exon	140	8	6	F	52
10H038	FLT3-Vb	chr13	28624359	28631497	novel_donor	13	6	4	F	18
10H038	FLT3-Vb	chr13	28624359	28631497	skipped_exon	130	6	4	F	18
11H027	FLT3-Va	chr13	28623598	28624232	novel_acceptor	76	8	6	F	31

11H027	FLT3-Va	chr13	28623598	28624232	skipped_exon	140	8	6	F	31
11H027	FLT3-Vb	chr13	28624359	28631497	novel_donor	13	6	4	F	9
11H027	FLT3-Vb	chr13	28624359	28631497	skipped_exon	130	6	4	F	9
11H027	NOTCH2-Vb	chr1	120484377	120493347	skipped_exon	273	18	15	T	5
11H160	FLT3-Va	chr13	28623598	28624232	novel_acceptor	76	8	6	F	34
11H160	FLT3-Va	chr13	28623598	28624232	skipped_exon	140	8	6	F	34
11H160	FLT3-Vb	chr13	28624359	28631497	novel_donor	13	6	4	F	5
11H160	FLT3-Vb	chr13	28624359	28631497	skipped_exon	130	6	4	F	5
11H160	NOTCH2-Vb	chr1	120484377	120493347	skipped_exon	273	18	15	T	13
04H048	CD13-Vc	chr15	90347848	90348353	novel_donor	44	5	4	F	4
04H048	FLT3-Va	chr13	28623598	28624232	novel_acceptor	76	8	6	F	16
04H048	FLT3-Va	chr13	28623598	28624232	skipped_exon	140	8	6	F	16
04H048	NOTCH2-Vb	chr1	120484377	120493347	skipped_exon	273	18	15	T	22
05H078	CD13-Vc	chr15	90347848	90348353	novel_donor	44	5	4	F	7
05H078	FLT3-Va	chr13	28623598	28624232	novel_acceptor	76	8	6	F	24
05H078	FLT3-Va	chr13	28623598	28624232	skipped_exon	140	8	6	F	24
05H078	NOTCH2-Vb	chr1	120484377	120493347	skipped_exon	273	18	15	T	6
07H020	CD13-Vc	chr15	90347848	90348353	novel_donor	44	5	4	F	19
07H020	FLT3-Va	chr13	28623598	28624232	novel_acceptor	76	8	6	F	14
07H020	FLT3-Va	chr13	28623598	28624232	skipped_exon	140	8	6	F	14
07H020	NOTCH2-Va	chr1	120497855	120506197	skipped_exon	111	13	11	T	12
07H020	NOTCH2-Vb	chr1	120484377	120493347	skipped_exon	273	18	15	T	32
08H048	CD13-Vc	chr15	90347848	90348353	novel_donor	44	5	4	F	12
08H048	FLT3-Va	chr13	28623598	28624232	novel_acceptor	76	8	6	F	33
08H048	FLT3-Va	chr13	28623598	28624232	skipped_exon	140	8	6	F	33
08H048	NOTCH2-Vb	chr1	120484377	120493347	skipped_exon	273	18	15	T	6
08H065	FLT3-Va	chr13	28623598	28624232	novel_acceptor	76	8	6	F	19
08H065	FLT3-Va	chr13	28623598	28624232	skipped_exon	140	8	6	F	19
08H082	FLT3-Va	chr13	28623598	28624232	novel_acceptor	76	8	6	F	33
08H082	FLT3-Va	chr13	28623598	28624232	skipped_exon	140	8	6	F	33
08H082	FLT3-Vb	chr13	28624359	28631497	novel_donor	13	6	4	F	9
08H082	FLT3-Vb	chr13	28624359	28631497	skipped_exon	130	6	4	F	9
08H082	NOTCH2-Vb	chr1	120484377	120493347	skipped_exon	273	18	15	T	21
10H089	CD13-Va	chr15	90347848	90348552	skipped_exon	140	5	3	F	15

10H089	CD13-Vc	chr15	90347848	90348353	novel_donor	44	5	4	F	16
10H089	FLT3-Va	chr13	28623598	28624232	novel_acceptor	76	8	6	F	13
10H089	FLT3-Va	chr13	28623598	28624232	skipped_exon	140	8	6	F	13
10H089	FLT3-Vb	chr13	28624359	28631497	novel_donor	13	6	4	F	7
10H089	FLT3-Vb	chr13	28624359	28631497	skipped_exon	130	6	4	F	7
10H089	NOTCH2-Va	chr1	120497855	120506197	skipped_exon	111	13	11	T	28
10H089	NOTCH2-Vb	chr1	120484377	120493347	skipped_exon	273	18	15	T	38
12H039	CD13-Vc	chr15	90347848	90348353	novel_donor	44	5	4	F	5
12H039	FLT3-Va	chr13	28623598	28624232	novel_acceptor	76	8	6	F	11
12H039	FLT3-Va	chr13	28623598	28624232	skipped_exon	140	8	6	F	11
12H039	FLT3-Vb	chr13	28624359	28631497	novel_donor	13	6	4	F	4
12H039	FLT3-Vb	chr13	28624359	28631497	skipped_exon	130	6	4	F	4
12H039	NOTCH2-Va	chr1	120497855	120506197	skipped_exon	111	13	11	T	8
12H039	NOTCH2-Vb	chr1	120484377	120493347	skipped_exon	273	18	15	T	28
12H175	FLT3-Va	chr13	28623598	28624232	novel_acceptor	76	8	6	F	12
12H175	FLT3-Va	chr13	28623598	28624232	skipped_exon	140	8	6	F	12
13H058	CD13-Vc	chr15	90347848	90348353	novel_donor	44	5	4	F	4
13H058	FLT3-Va	chr13	28623598	28624232	novel_acceptor	76	8	6	F	11
13H058	FLT3-Va	chr13	28623598	28624232	skipped_exon	140	8	6	F	11
13H058	FLT3-Vb	chr13	28624359	28631497	novel_donor	13	6	4	F	4
13H058	FLT3-Vb	chr13	28624359	28631497	skipped_exon	130	6	4	F	4
13H058	NOTCH2-Vb	chr1	120484377	120493347	skipped_exon	273	18	15	T	19
09H106	CD13-Va	chr15	90347848	90348552	skipped_exon	140	5	3	F	8
09H106	CD13-Vc	chr15	90347848	90348353	novel_donor	44	5	4	F	15
09H106	FLT3-Va	chr13	28623598	28624232	novel_acceptor	76	8	6	F	24
09H106	FLT3-Va	chr13	28623598	28624232	skipped_exon	140	8	6	F	24
09H106	NOTCH2-Va	chr1	120497855	120506197	skipped_exon	111	13	11	T	13
09H106	NOTCH2-Vb	chr1	120484377	120493347	skipped_exon	273	18	15	T	35

cohorts:
CBF-AML
MLL-F
NUP98-ND1
CEBPA
MLL-PTD
